# Hypoxia-induced cysteine metabolism reprogramming is crucial for the tumorigenesis of colorectal cancer

**DOI:** 10.1016/j.redox.2024.103286

**Published:** 2024-07-26

**Authors:** Zhang Lin, Shiyi Yang, Qianqian Qiu, Gaoping Cui, Yanhua Zhang, Meilian Yao, Xiangyu Li, Chengkun Chen, Jun Gu, Ting Wang, Peng Yin, Longci Sun, Yujun Hao

**Affiliations:** a State Key Laboratory of Systems Medicine for Cancer, Shanghai Cancer Institute, Renji Hospital, School of Biomedical Engineering, School of Medicine, Shanghai Jiao Tong University, Shanghai, 200032, China; b Department of Medical Oncology, Shanghai Pulmonary Hospital, Tongji University Medical School Cancer Institute, Tongji University School of Medicine, Shanghai, 200433, China; c Key Laboratory of Chemical Biology and Traditional Chinese Medicine Research, College of Chemistry and Chemical Engineering, Hunan Normal University, Changsha, 410081, China; d Department of Gastrointestinal Surgery, Renji Hospital, School of Medicine, Shanghai Jiao Tong University, Shanghai, 200127, China

**Keywords:** Colorectal cancer, Cysteine/cystine, Transporter genes, ATF4, Hypoxia, ROS homeostasis

## Abstract

Metabolic reprogramming is a hallmark of human cancer, and cancer-specific metabolism provides opportunities for cancer diagnosis, prognosis, and treatment. However, the underlying mechanisms by which metabolic pathways affect the initiation and progression of colorectal cancer (CRC) remain largely unknown. Here, we demonstrate that cysteine is highly enriched in colorectal tumors compared to adjacent non-tumor tissues, thereby promoting tumorigenesis of CRC. Synchronously importing both cysteine and cystine in colorectal cancer cells is necessary to maintain intracellular cysteine levels. Hypoxia-induced reactive oxygen species (ROS) and ER stress regulate the co-upregulation of genes encoding cystine transporters (SLC7A11, SLC3A2) and genes encoding cysteine transporters (SLC1A4, SLC1A5) through the transcription factor ATF4. Furthermore, the metabolic flux from cysteine to reduced glutathione (GSH), which is critical to support CRC growth, is increased due to overexpression of glutathione synthetase GSS in CRC. Depletion of cystine/cysteine by recombinant cyst(e)inase effectively inhibits the growth of colorectal tumors by inducing autophagy in colorectal cancer cells through mTOR-ULK signaling axis. This study demonstrates the underlying mechanisms of cysteine metabolism in tumorigenesis of CRC, and evaluates the potential of cysteine metabolism as a biomarker or a therapeutic target for CRC.

## Highlights

• Cysteine enrichment is a key signature of CRC.
• Synchronously importing both cysteine and cystine is critical for CRC.
• Hypoxia induces the expression of both cystine and cysteine transporters through ATF4.
• The flux from cysteine to GSH is enhanced in CRC by GSS overexpression.
• Depleting cysteine/cystine inhibits CRC growth via mTOR-ULK1 mediated autophagy.

## Graphical abstract

**Figure:**
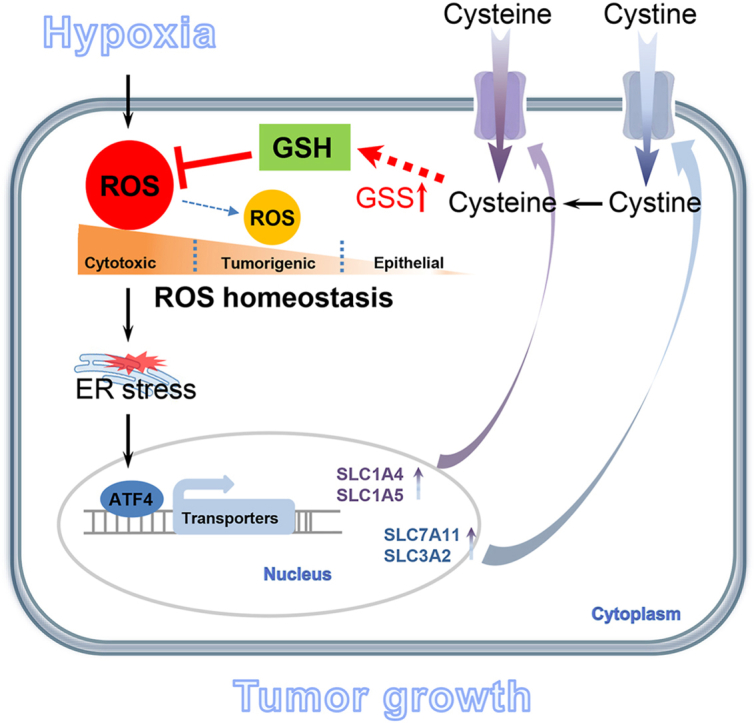

## Abbreviations:

CRC: Colorectal cancer
ROS: Reactive oxygen species
ER stress: Endoplasmic reticulum stress
GSH: Glutathione
DEMs: Differentially expressed metabolites
PCA: Principal component analysis
OPLS-DA: Orthogonal partial least squares-discriminant analysis
*CAKP*: *CDX2P-CreER* ^*T2*^ *; Apc* ^*15lox/+*^ *; Kras* ^*LSL-G12D*^ *; R26-Pik3ca* ^*H1047R*^
ISR: Integrated stress response
Tg: Thapsigargin
DEGs: Differentially expressed genes
NAC: N-acetyl cysteine
NEAA: Non-essential amino acids
GSS: Glutathione synthetase
CDD: Cystine/cysteine deficient diet
GC-TOF MS: Gas chromatography time-of-flight mass spectrometry
CE-TOF MS: Capillary electrophoresis time-of-flight mass spectrometry
^1^H HRMAS NMR: ^1^H high resolution magic-angle spinning nuclear magnetic resonance
LC-MS: Liquid chromatography-mass spectrometry

## 1 Introduction

Cancer cells exhibit abnormal metabolic pathways to support their survival, growth, and metastasis, and metabolic reprogramming becomes one of the hallmarks of human cancer [1,2]. Understanding the tumor-specific metabolism provides important opportunities for the diagnosis, prognosis, and treatment of human cancer [2,3]. Colorectal cancer (CRC) is the third most commonly occurring cancer and the second leading cause of death from cancer worldwide in 2020 [4]. Several risk factors, including genetic alteration, environment and lifestyle, or other diseases (inflammatory bowel disease, obesity, diabetes et al.), contribute to the initiation and progression of CRC [5]. The alterations in metabolic pathways also play important roles in CRC tumorigenesis [6]. The Wnt signaling pathway regulates the expression of pyruvate dehydrogenase kinase 1 (PDK1) or lactate transporter MCT-1 (SLC16A1) to promote the proliferation or angiogenesis of colorectal tumors [7,8]. KRAS mutations upregulate the expression of glucose transporter GLUT1 to enhance glucose uptake [9] or asparagine synthetase ASNS to adapt cells under glutamine deficient condition in CRC [10]. PIK3CA mutations upregulate the expression of glutamic-pyruvic transaminase 2 (GPT2) through PDK1-ATF4 axis, therefore increasing the flux from glutamine to TCA cycle for ATP generation [11,12]. Those studies describe the metabolic pathways which are regulated by some genetic alterations or signaling pathways in CRC. However, the common metabolic signatures in CRC tumors and their underlying mechanisms are still not fully understood.

Metabolome analysis is a crucial approach to explore cancer metabolism. Metabolomic analyses reveal that CRC shows metabolic alterations in both adenoma and adenocarcinoma, including glycolysis, one-carbon metabolism, amino acids metabolism, and lipid metabolism [6,13]. It is worth noting that the profiles of differentially expressed metabolites (DEMs) varied from different studies due to the study population, tumor heterogeneity, sample preparation, or analysis platform [[13], [14], [15], [16], [17], [18], [19]]. Therefore, screening for common DEMs across multiple studies will help us to elucidate the pivotal metabolic alterations in CRC and, moreover, provide opportunities for the diagnosis, prognosis, or treatment of CRC.

Cysteine is a non-essential amino acid which becomes essential for cancer cells [20]. Cystine (oxidized cysteine dimer) is critical for the growth of EGFR or BRAF mutant human mammary epithelial cells, as well as EGFR mutant non-small-cell lung cancer [21]. Wu et al. show that cystine/cysteine support CRC growth through mTOR pathway [22]. Tarragó-Celada et al. report that cysteine metabolism is targetable vulnerability of metastatic colorectal cancer [23]. Normally, in cancer cells, intracellular cysteine relies on cystine uptake by cystine/glutamate antiporter xCT [24,25]. However, chronic lymphocytic leukemia cells are able to transport cysteine but not cystine [26], and pancreatic ductal adenocarcinoma (PDAC) cells transport cysteine to compensate for the intracellular cysteine pool once xCT/SLC7A11 is knocked out [27,28], suggesting that the underlying mechanisms of cysteine metabolism in various cancer types are different.

In this study, cysteine is identified as one of the most common enriched metabolites in CRC compared with paired normal colon tissues. Both cystine and cysteine uptake are crucial to maintain high intracellular cysteine levels, therefore supporting CRC growth. We further elucidate the mechanisms of cysteine metabolism reprogramming in colorectal cancer.

## 2 Materials and methods

### 2.1 Human tissues collection

This study was approved by the Ethics Committee at Renji Hospital, Shanghai Jiao Tong University School of Medicine (Shanghai, China). Informed consents were obtained from all participants. Human tissue samples were collected from colorectal cancer (CRC) patients at Shanghai Renji Hospital since 2018 to 2022.20 (Cohort 1) and 40 (Cohort 2) patients contributed paired CRC tissue samples and adjacent non-tumor tissue samples. All samples were collected within 20 min after the surgery and immediately frozen at −80 °C for metabolomic, RNA-seq analyses or Western blots.

### 2.2 Genetically engineered mice

The animal experiments were performed in accordance with protocols approved by the IACUC committee at Shanghai Cancer Institute, Renji Hospital, Shanghai Jiao Tong University School of Medicine. Mice strains of *CDX2P-CreER*^*T2*^ (022,390); *Apc*^*15lox/+*^ (029,275)*; Kras*^*LSL-G12D*^ (008179)*; R26-Pik3ca*^*H1047R*^ (016,977) were purchased from The Jackson Laboratory. As *R26-Pik3ca*^*H1047R*^ (016,977) is FVB background but the other three mouse strains are C57BL/6 background, *R26-Pik3ca*^*H1047R*^ mice were backcrossed to C57BL/6 mice for 10 generation (F10) to get *R26-Pik3ca*^*H1047R*^ strain as close to pure C57BL/6 genetic background as possible. Then *CDX2P-CreER*^*T2*^ mice were crossed with *Apc*^*15lox/+*^ mice to get *CDX2P-CreER*^*T2*^*;Apc*^*15lox/15lox*^ mice, and *Kras*^*LSL-G12D*^ mice were bred with *R26-Pik3ca*^*H1047R*^ (B6) to get *Kras*^*LSL-G12D*^*;R26-Pik3ca*^*H1047R*^ mice. *CDX2P-CreER*^*T2*^*;Apc*^*15lox/15lox*^ mice and *Kras*^*LSL-G12D*^*;R26-Pik3ca*^*H1047R*^ mice were crossed to get *CDX2P-CreER*^*T2*^*;Apc*^*15lox/+*^*;Kras*^*LSL-G12D*^*;R26-Pik3ca*^*H1047R*^ strain. Cre-mediated the deletion of *Apc* allele with loxP-flanked *Apc* exon 15 and the expression of *Kras*^G12D^ and *Pik3ca*^H1047R^ were achieved by injection with tamoxifen (100 mg/kg body weight; Sigma-Aldrich, St Louis, MO) once daily for 5 consecutive days intraperitoneally. For stable-isotope tracing experiments, the mice were infused with ^13^C_3_-cysteine to examine the cysteine flux in colorectal tumors two months post tamoxifen administration. For cyst(e)inase treatment experiments, 4 weeks post tamoxifen administration, mice were randomly assigned into two groups and treated with vehicle or cyst(e)inase (50 mg/kg) respectively for 3 times a week.

### 2.3 Cell culture

CRC cell lines HCT116, DLD1, RKO, HT29, SW480, LOVO (metastatic origin), HCT-8, LS174T and normal human colon epithelial cells (NCM460, FHC) were purchased from American Type Culture Collection. CRC cell lines were maintained in McCoy's 5 A medium (Gibco). NCM460 and FHC were maintained in DMEM medium (Sigma). All cell lines were maintained at 37 °C in a 5 % CO_2_ atmosphere with medium supplemented with 10 % fetal bovine serum (FBS, Gibco) and 1 % penicillin-streptomycin (Gibco). Cell culture medium was routinely assayed for mycoplasma to ensure that they were mycoplasma-free (Yeasen). The cell lines were authenticated by the Shanghai Bowing Biotech (Shanghai, China) using STR profiling. To make cystine/cysteine deficient medium, DMEM medium without cyst(e)ine, methionine, and glutamine (Gibco, Cat# 21,013,024) were supplied with 2 mM glutamine and 200 μM methionine.

### 2.4 Reagents

l-cysteine, l-cystine and l-methionine were purchased from Shanghai Sangon (Shanghai, China). ^13^C_2_-l-cystine and ^13^C_3_-l-cysteine were purchased from Cambridge Isotope Laboratory (MA, USA). HPLC grade methanol, acetonitrile, chloroform, hexane and formic acid were purchased from Merck Chemicals (Darmstadt, Germany). Pyridine, methoxyamine HCl, 2-chloro-l-phenylalanine, heptadecanoic acid, MTBSTFA (1 % t-BDMCS), glutamine, N-acetyl cysteine (NAC), reduced glutathione (GSH), dimethyl α-ketoglutarate, cystathione, homocysteine, nucleosides and anhydrous sodium sulfate were purchased from Sigma Aldrich (MO, USA). MSTFA (N-methyl-N-(trimethylsilyl)trifluoroacetamide) with 1 % trimethylchlorosilane and 1 % TMCS was purchased from Thermo-Fisher Scientific (NJ, USA). Ultrapure water was produced by a Mill-Q Reference system equipped with a LC-MS Pak filter (Millipore, MA, USA). Non-essential amino acids (NEAA) and pyruvate was obtained from Invitrogen.

### 2.5 Untargeted metabolomic profiling

The metabolic profiles of paired colorectal tumor and adjacent non-tumor tissues were analyzed by Metabolic-Profile Biotechnology (Shanghai, China). Briefly, 50 mg of tissues were minced with liquid nitrogen, and put in 250 μL mixture of chloroform, methanol, and water (2:5:2). The samples were then placed at −20 °C for 20 min to extract metabolites, followed by centrifugation at 12,000 rpm for 10 min. The upper supernatants were transferred to a glass vial with heptadecanoic acid (20 μg/ml) as internal standard, and then vacuum dried at room temperature. The metabolites were chemically derivatized with 80 μL of methoxyamine (15 mg/ml in pyridine) at 30 °C for 90 min, followed by adding 80 μL of BSTFA (1 % TMCS) at 70 °C for 60 min. The untargeted metabolomic profiling were conducted on a time-of-flight mass spectrometry (TOF MS) system (Pegasus HT, Leco Corp., St. Joseph, MO, USA) with an Agilent 7890 gas chromatography. A Rxi-5ms capillary column (30 m × 250 μm i.d., 0.25-μm film thickness; Restek corporation, Bellefonte, PA, USA) was used for separation. Data were acquired with *m*/*z* range of 30–600 at an acquisition rate of 20 spectra per second. The acquired raw MS files were processed in ChromaTOF software (v3.30, Leco Co., CA) and MATLAB 7.0. Principal component analysis (PCA) and orthogonal partial least squares discriminant analysis (OPLS-DA) were carried out to differentiate tumors and adjacent normal tissues by SIMCA-P 12.0 Software package (Umetrics, Sweden). The differentially expressed metabolites (DEMs) are listed in Supplementary Table S1.

The metabolic profiles of HCT116 cells cultured with or without cystine/cysteine were analyzed by Biotree Biotechnology (Shanghai, China). For cell sample preparation, 3 × 10^6^ cells were plated in 10 cm dishes. When reached 70 % confluency, cells were washed with PBS and changed to cystine/cysteine deficient medium with or without 100 μM cystine and 200 μM cysteine. After 24 h, cells were collected with 1 ml pre-chilled methanol containing 2-chloro-l-phenylalanine (2 μg/ml) as internal standard and metabolites were extracted by sonication. After removing the debris by centrifugation at 12,000 for 15 min, the supernatants were dry with nitrogen gas. Then, the dried samples were reconstituted in 200 μL of 50 % acetonitrile by sonication on ice for 10 min. The constitution was then centrifuged at 12,000 rpm for 15 min at 4 °C, and 75 μL of supernatant was transferred to a fresh glass vial for untargeted LC-TOFMS analysis in Biotree Biotechnology (Shanghai, China). The LC separation was carried out using a 1290 Infinity series UHPLC System (Agilent Technologies), equipped with a UPLC BEH Amide column (2.1 × 100 mm, 1.7 μm, Waters). The TripleTOF 6600 mass spectrometry (AB Sciex) was used to acquire MS/MS spectra on an information-dependent basis (IDA) mode. Sample detection was performed in both positive and negative mode of electrospray ionization (ESI) source.

### 2.6 Targeted metabolomic analysis

3 × 10^6^ cells were plated in 10 cm dishes. When cells were reached 70 % confluency, cells were washed with PBS and changed to cystine/cysteine deficient medium overnight. Then 100 μM cystine and 200 μM cysteine were supplied into cystine/cysteine deficient medium to treat cells for 2 h. Cells were then quenched with 1 ml pre-chilled methanol containing 2-chloro-l-phenylalanine (2 μg/ml) as internal standard, and metabolites were extracted as described above. To analyze metabolites of cells after gene silencing, control siRNA or siRNAs for target genes were transfected into cells. 48 h post-transfection, cells were washed with PBS and change to cystine/cysteine deficient medium overnight. The metabolites were extracted as described above and analyzed with Agilent 1290 UHPLC system coupled to an AB QTRAP 6500 (AB SCIEX, USA). A Waters ACQUITY UPLC BEH Amide column (particle size, 1.7 μm; 100 mm (length) × 2.1 mm (i.d.)) was used for separation. The mass spectrometer was detected in multiple reaction monitoring (MRM) mode. The MRM transitions (*m*/*z*), DPs (V), and CEs (V) of target metabolites are listed in Supplementary Table S2. MRM data were acquired using Analyst 1.6.1 software (Applied Biosystems SCIEX). Chromatogram review and peak area integration were conducted using SCIEX OS (Version 1.5.0.23389, Applied Biosystems SCIEX) software. The abundances of metabolites were presented as the peak area ratio of targeted metabolites versus internal standard 2-chloro-l-phenylalanine of each sample.

### 2.7 Mouse infusion

Surgical procedures for mouse infusion were similar as described previously [12]. In brief, mice were anesthetized and made a 2 cm skin incision on the right side of the neck. The jugular vein was isolated with blunt forceps and tied on both proximal and distal ends of the vessel. RenaSil Silicone Rubber Tubing (0.025″ OD × 0.012″ID) (BioLabs, German) was inserted into the vein. The ends of the free catheter were tunneled under the skin to the back of the neck and sealed with steel plugs. One day after surgery, mice were infused by bolus (10 μM ^13^C_3_-cysteine in 150 mM NaCl solution) as 0.3ml/20 g mice first. Then solution (10 μM ^13^C_3_-cysteine in 150 mM NaCl solution) at a rate of 0.3 ml/h/20 g were constantly infused into the mice by a micropump (Longer, England). Mice were sacrificed 4 h later, and serum, tumors and adjacent normal tissues were collected and frozen at −80 °C for metabolites analysis.

### 2.8 Stable isotope tracing experiments

For cell samples, 3 × 10^6^ cells were plated in 10 cm dishes. When cells were reached ∼90 % confluency, cells were washed with PBS and change to cystine/cysteine deficient medium supplemented with either 100 μM ^13^C_2_-cystine or 100 μM ^13^C_3_-cysteine, then followed by metabolites extraction. The metabolites extracting buffer was pre-chilled methanol plus 2-chloro-l-phenylalanine (2 μg/ml) and heptadecanoic acid (20 μg/ml) as internal standard. 1 ml extracting buffer/1 × 10^7^ cells were directly added on the cells at the plate surface. Tissue samples were lysed with a TissueLyser II (Qiagen, Germany) with 1 ml extracting buffer per 100 mg tissue. Serum samples were lysed by directly adding 3 folds extracting buffer. The debris were discarded after centrifugation by 12,000 rpm for 15 min at 4 °C. The supernatants were applied for mass spectrometry assay.

GC-MS was applied to trace cysteine and taurine as described previously [11]. Briefly, the supernatant was dried with CentriVap vaccum concentrate (Labconco, Missouri, USA). TBDMS (MTBSTFA + TBDMCS, REGIS Technologies):Acetonitrile (2:1) were used for derivatization of metabolites at 65 °C for 1 h 1 μl of samples was injected into GC-MS (Agilent Technologies) for analysis. The *m*/*z* of C13 labeled cysteine was M0 (406), M1 (407), M2 (408) and M3 (409), and *m*/*z* of C13 labeled taurine was M0 (296), M1 (297), M2 (298). The relative abundance of unlabeled or labeled metabolites was calculated by normalizing to internal standard. For the fraction of C13 labeled metabolites, the total pool of each metabolite was set to 100 %, and C13 labeled metabolites isotopomer distribution (enrichment) indicated percentage of each isotopomer to total pool.

LC-MS was applied to trace reduced glutathione (GSH). Briefly, 3 μl of the supernatant was directly injected into UHPLC-QTOF/MS for analysis. The *m*/*z* of C13 labeled glutathione was M0 (308.09) to M10 (318.09). For the analysis of the fraction of C13 labeled metabolites, the total pool of each metabolite was set as 100 %, and C13 labeled metabolites isotopomer distribution (enrichment) indicated percentage of each isotopomer to total pool.

### 2.9 Gene silencing

The siRNAs targeting indicated genes and scramble siRNA were synthesized by Shanghai Biotech (Shanghai, China). To knockdown the designated genes, three independent siRNAs were mixed (similar as esiRNA strategy), and then the mixture was transfected into cells with lipofectamine 3000 according to manufacturer's instructions. 48–72 h post-transfection, cells were conducted for various assays. The shRNA lentivirus targeting SLC7A11, SLC1A5, ATF4, PERK, and a non-targeting control (NC) were purchased from GeneCopoeia (Guangzhou, China). Cells were infected with lentivirus and screened for stable pools with puromycin (Invitrogen) (0.8 μg/ml for HCT116 and 2.0 μg/ml for DLD1) for 2 weeks. The knockdown efficacy was validated by qRT-PCR and Western blot analyses. The sequences of siRNA and shRNA are listed in Supplementary Table S3.

### 2.10 Quantitative real-time PCR

Total RNA was extracted using RNAfast200 kits (Fasted, Shanghai, China) according to the manufacturer's instructions and the quality of isolated RNAs were determined by Nanodrop (Thermo Scientific, Massachusetts, USA). Reverse transcription (RT) was conducted by PrimeScript RT Master Mix (TaKaRa, Kusatsu, Japan). cDNAs were used as templates for PCR. Real-time PCR was carried out using ChamQTM SYBR® Color qPCR Master Mix (Vazyme, Nanjing, China). ATCB (β-actin) was applied to normalize genes' expression. The qRT-PCR primers are listed in Table S3.

### 2.11 Immunoblotting

Cells were lysed in RIPA buffer (invitrogen) supplemented with complete Protease Inhibitor (Roche) and PhosSTOP (Roche). Cell lysates were cleared by centrifugation (12,000 rpm, 4 °C, 15 min) and boiled with 4 × loading buffer (Tanon, Shanghai, China) at 100 °C for 5 min. Equal amounts of total protein were subjected to SDS-PAGE and then electrically transferred in constant current of 200 mA for 2 h to a 0.45 μm PVDF membrane (Millipore). After blocked with 5 % nonfat-dried Milk at room temperature for 1 h, the PVDF membrane was incubated with primary antibodies at 4 °C overnight and then secondary antibodies at room temperature for 2 h. Finally, the blots were detected by adding HRP substrate (Millipore) and visualized using UVP ChemStudio/PLUS Imaging System (Analytik Jena). The antibodies used in this study are listed in Supplementary Table S4.

### 2.12 Cell proliferation assays

For cystine or cysteine treatment, cells were plated in 24-well plates at 5000 cells per well in complete DMEM medium. After 24 h, cells were washed with PBS and changed to cystine deficient DMEM medium (Gibco, 21,013,024) supplemented with 5 % dialysed FBS (Gibco, 30067334), 2 mM glutamine and 200 μM methionine. Indicated concentrations of cysteine or cystine were added to different wells in triplicate. 72 h later, cells (including floating cells in medium) were collected with centrifugation (5000 rpm, 5 min) and counted with hemocytometer by Trypan-Blue exclusive assay.

For cell proliferation after gene editing, 2000 cells per well were seeded in a 96-well plate in complete DMEM medium. 24 h later, cells were starved with cystine/cysteine deficient medium overnight, and then changed to medium containing 100 μM cystine and 100 μM cysteine. Cell Counting Kit-8 (Dojindo, Japan) were applied for 5 consecutive days to measure viable cells according to the manufacturer's instructions. Absorbance at OD450 was plotted for cell growth curves.

For cytotoxic assay, 10,000 cells were seeded in a 24-well plate in complete DMEM medium. 24 h later, cells were changed to cystine/cysteine deficient medium (-cyst(e)ine) or cystine/cysteine abundant (100 μM cystine and 200 μM cysteine) medium (+cyst(e)ine). 72 h later, viable cells were collected and counted with hemocytometer by Trypan-Blue exclusive assay. Relative survival was calculated by -cyst(e)ine/+cyst(e)ine × 100 %. For 96-well plate, 5000 cells per well were seeded, and the indicated drugs were added after 24 h 48 h later, viable cells were measured using Cell Counting Kit-8. Relative survival was calculated by treatment OD450/vehicle OD450 × 100 %.

### 2.13 Luciferase reporter assay

The promoters of SLC1A4, SLC1A5, SLC7A11 or SLC3A2 were amplified by reverse transcription PCR with genomic DNA of HCT116 cells and cloned into pGL3-Basic vector respectively. Indicated pGL3-promoter constructs and internal control Renilla LUC were co-transfected into two sets of HCT116 cells. 48 h post-transfection, one set of cells was incubated in hypoxia condition overnight (1 % O_2_, 5 % CO_2_, 94 % N_2_) and the other set was kept in normoxia condition overnight. The luciferase reporter assay was conducted using a Dual-Luciferase Reporter Assay System (Promega, E1910) according to the manufacturer's instructions. Luminescence was measured using a Gen 5 microplate reader (BIOTEK, USA). Relative luciferase activity was present as the ratio of hypoxia luciferase reading versus normoxia luciferase reading.

### 2.14 Chromatin immunoprecipitation PCR (ChIP-PCR)

HCT116 cells were treated with or without H_2_O_2_ overnight. ChIP assays were performed using the ChIP Assay Kit (Beyotime, #P2078, Shanghai, China) according to the manufacturer's instructions. Briefly, cells were cross-linked with a final concentration of 1 % formaldehyde in growth medium for 10 min at 37 °C and quenched by the addition of glycine solution for 5 min at room temperature (RT). Then cells were harvested, lysed, and sonicated. Protein A + G Agarose/Salmon Sperm DNA were used to preclear the whole cell lysate for 30 min at 4 °C. After collecting input samples, the supernatants were divided into two parts and incubated with ATF4 or control IgG antibodies at 4 °C overnight, followed by Protein A + G Agarose/Salmon Sperm DNA incubation. Thereafter, the beads were washed sequentially with Low-Salt Immune Complex Wash Buffer, High-Salt Immune Complex Wash Buffer, LiCl Immune Complex Wash Buffer and TE Buffer (twice). DNA-protein complexes were eluted with elution buffer (1 % SDS and 0.1 M NaHCO_3_), de-crosslinked by adding 0.2 M NaCl, and heated for 4 h at 65 °C. Then, the proteins were digested with proteinase K for 1 h at 45 °C, and the DNA segments were purified by a DNA Purification Kit (D0033, Beyotime Biotechnology, Shanghai, China) for qPCR reaction. The primers for ChIP-PCR and ATF4 binding sites sequence are listed in Supplementary Table S3.

### 2.15 Recombinant cyst(e)inase purification

The recombinant cyst(e)inase was constructed and purified as described previously [47]. Briefly, CTH (Cystathionine gamma-lyase) cDNA was cloned into pET28a, and mutated as E59T and E339V by site-directed mutagenesis. pET28a-CTH-E59T-E339V plasmids were transformed into BL21 (DE3) bacterial strain and recombinant His-tagged cyst(e)inase was purified by Ni-NTA beads according to Qiagen handbook (Qiagen, Germany). Recombinant cyst(e)inase was conjugated to lysyl residues using methoxy PEG succinimidyl carboxymethyl ester of MW 5000 (PEG-5K, JenKem Technology or NOF America Corporation) to prevent renal clearance and impart long circulation persistence.

### 2.16 Xenograft

Animal experiments were approved by the Animal Ethics Committee at Shanghai Cancer Institute, Renji Hospital, Shanghai Jiao Tong University School of Medicine. Female nude mice with 4–6 weeks old were kept in SPF conditions one week before receiving xenograft. Tumor volume was measured at the indicated time points and calculated as length × width [2]/2.

To verify the tumor-promoting effect of cysteine in vivo, HCT116 cells were firstly injected subcutaneously into both flanks of mouse at 3 × 10^6^ cells in 100 μL PBS. When the average diameter of tumors reached ∼5 mm, mice were randomly divided into cysteine treatment group (n = 4) or control group (n = 4). The mice were intraperitoneally injected with PBS vehicle or 100 μL cysteine solution (50 mg/kg) 5 days a week for 4 weeks.

To elucidate in vivo tumor growth with cells after gene editing, 3 × 10^6^ cells for each cell clones were injected subcutaneously and bilaterally into athymic nude mice and tumor size and/or weight were measured. Briefly, for stable cell clones, cells were directly injected into flanks. For transient transfected cells, indicated siRNAs were transfected into specified cell clones, and cells were collected to generate subcutaneous tumors three days post-transfection.

To explore the effect of cyst(e)inase on xenograft tumors, HCT116 cells or two pieces of patient-derived xenograft tumors (∼2–4 mm^3^) were injected subcutaneously and bilaterally into athymic nude mice. Once tumor sizes reached 100–150 mm^3^, mice were randomly assigned into two groups (5–6 mice per group) and treated with vehicle or cyst(e)inase (50 mg/kg) respectively for 3 times a week.

### 2.17 Transcriptome sequencing

Total RNAs of tissues or cells were extracted from cells or paired colorectal tumor and adjacent non-tumor tissues using TRIzol® Reagent (Invitrogen) according to the manufacturer's instructions and genomic DNA was removed using DNase I (TaKara). Then RNA quality was determined by 2100 Bioanalyser (Agilent Technologies) and quantified using the ND-2000 (NanoDrop Technologies). RNA quality was determined by 2100 Bioanalyser (Agilent Technologies) and quantified using the ND-2000 (NanoDrop Technologies) before used to construct sequencing library. RNA purification, reverse transcription, library construction and sequencing were performed at Shanghai Majorbio Bio-pharm Biotechnology Co., Ltd. (Shanghai, China).

To identify different expressed genes (DEGs), the expression level of each gene was calculated according to FPKM. The expression of genes with |log_2_ (fold change)| ≥ 1 and p value ≤ 0.01 between two groups were considered as DEGs. In addition, functional-enrichment analysis including GO and KEGG were carried out by Genetools (https://github.com/tanghaibao/Goatools) and KOBAS (http://kobas.cbi.pku.edu.cn/home.do).

### 2.18 Fluorescent LC3 puncta

HCT116 or DLD1 cells were transduced with the lentivirus expressing mCherry-EGFP-LC3B fusion protein (GeneCopoeia, Guangzhou, China) at 15 MOI, and stable mCherry-EGFP-LC3B cells were selected with puromycin (Invitrogen) (0.8 μg/ml for HCT116 and 2.0 μg/ml for DLD1). After treated with cystine/cysteine depletion condition or cyst(e)inase, mCherry-EGFP-LC3B cells were washed with PBS and then fixed 4 % paraformaldehyde. Fluorescent puncta pictures were captured with a confocal laser scanning microscope. For each well, the number of GFP and mCherry dots was determined by three independent views. DAPI was used for nuclei staining.

### 2.19 Statistical analyses

Most data were presented as mean ± SD and xenograft tumor growth curves were presented as mean ± SEM. Student's t-test was applied for the comparation between two groups, and two-way ANOVA was used for tumor growth and cell growth curves by GraphPad Prism 5.0 Software. P < 0.05 was considered statistically significant.

## 3 Results

### 3.1 Cysteine is enriched in colorectal tumors

**Fig. 1:**
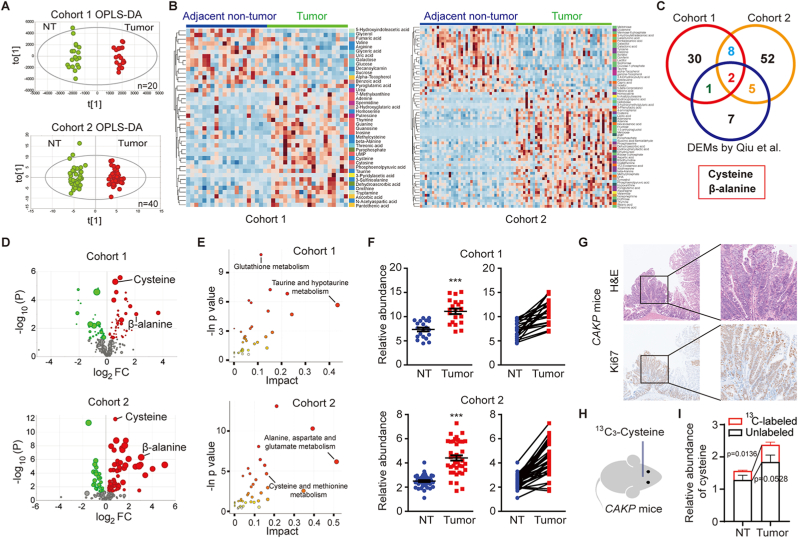
**Cysteine is enriched in colorectal tumors**. (A) OPLS-DA models for metabolites profiling of tumors (Tumor) and adjacent non-tumor samples (NT) from CRC patients by GC-TOF-MS (cohort 1, n = 20; cohort 2, n = 40). (B) Heatmaps of 41 and 67 differentially expressed metabolites (DEMs) identified from cohort 1 and cohort 2 respectively. (C) Venn diagram of DEMs in different cohorts between colorectal tumors and corresponding adjacent non-tumor tissues. Cohort 1 and cohort 2 were as described in (A). DEMs by Qiu et al. indicated the DEMs which were identified with 376 surgical specimens of CRC from four different hospitals with similar analysis platform [16]. (D) Volcano plots of DEMs from cohort 1 and cohort 2. Y axis represents -log_10_ (p value) between tumors and non-tumor tissues. X axis represents log_2_ (fold change) of tumors versus non-tumor tissues. The bubble size represents variable importance in the projection (VIP) value which reflects the contribution of the metabolites to differentiate tumors and non-tumor tissues. (E) KEGG pathway analysis of DEMs from cohort 1 and cohort 2. Bubble chart shows the enrichment of DEMs in metabolic pathways. Y axis represents -ln (p value) of pathway analysis. X axis represents impact factor (amount of DEMs enriched in the pathway/amount of all metabolites in the pathway). (F) l-cysteine was consistently increased in over 95 % tumors compared to their paired non-tumor tissues in both cohort 1 and cohort 2. (G) The H&E and Ki67 staining images of spontaneous colon tumors which were developed at two months post tamoxifen administration in a genetically engineered mouse model *CDX2P-CreER*^*T2*^*;Apc*^*15lox/+*^*;Kras*^*LSL-G12D*^*;R26-Pik3ca*^*H1047R*^ (*CAKP* mice). (H) ^13^C_3_-l-cysteine was infused in *CAKP* mice harboring spontaneous colon tumors. (I) Cysteine was enriched in CRC (Tumor) compared with adjacent non-tumor tissues (NT) in mice. Both ^13^C-labeled (red) and unlabeled (black) cysteine were measured, and relative abundance (peak area normalized to internal standard) were present (n = 8). The stacked column suggests total amount of cysteine, and ^13^C-labeled (red) column suggests the exogenous cysteine uptake specifically. Student's t-test was applied. Data are presented as mean ± SD. **p < 0.01; ***p < 0.001; ****p < 0.0001. (For interpretation of the references to color in this figure legend, the reader is referred to the Web version of this article.)

To identify the differentially expressed metabolites (DEMs) of colorectal tumors and adjacent non-tumor tissues, two independent cohorts of tissue samples were collected from colorectal cancer (CRC) patients at Shanghai Renji Hospital from 2018 to 2022. The differences between the metabolite profiles of CRC tumor samples and adjacent non-tumor samples were observed from the unsupervised principal component analysis (PCA) score plot (Fig. S1A). With a supervised PLS-DA (OPLS-DA) model, tumor samples and adjacent non-tumor samples achieved more significant separation (Fig. 1A), and permutation tests showed that OPLS-DA models were well fitted (Fig. S1B). Total 41, or 67 DEMs, were identified from cohort 1 or cohort 2 respectively (Fig. 1B and C, Table S1). With similar analytical platforms, Qiu et al. reported a panel of 15 significantly altered metabolites in colorectal tumors [16]. To discover the most consistent metabolic signature of CRC, integrated analyses with our DEMs and these 15 metabolites were performed. l-cysteine and β-alanine were consistently enriched in CRC in all these cohorts (Fig. 1C and D). With KEGG pathway analysis, DEMs from cohort 1 and cohort 2 were enriched in several cysteine-related metabolic pathways, including glutathione, methionine, taurine, and amino acids pathways (Fig. 1E and S1C). Indeed, in both cohort 1 and cohort 2, l-cysteine was consistently increased in over 95 % of tumors compared to their paired non-tumor tissues, whereas the abundance of other cysteine-related metabolites varied across different cohorts (Fig. 1F and S1D), suggesting that l-cysteine enrichment could serve as a key signature of CRC.

As a semi-essential amino acid, a small amount of l-cysteine can be generated by cells, but large amounts depend on exogenous uptake [20]. To determine whether the enrichment of l-cysteine in CRC was due to exogenous uptake, we developed spontaneous colon tumors with a genetically engineered mouse model, *CDX2P-CreER*^*T2*^*; Apc*^*15lox/+*^*; Kras*^*LSL-G12D*^*; R26-Pik3ca*^*H1047R*^ (*CAKP* mice) (Fig. 1G), and then infused [^13^C_3_]-l-cysteine in mice when advanced colon tumors occurred (Fig. 1G and H). Similar to the results obtained in human samples, the total amount of l-cysteine was higher in CRC compared with normal colon tissues of mice (Fig. 1I). Moreover, colon tumors contained much more ^13^C-labeled cysteine than normal colon tissues (Fig. 1I), suggesting cysteine uptake is higher in CRC. 10.13039/100014337Furthermore, consistent with previous reports [22], supplying with exogenous cysteine or cystine supported the growth of CRC cells, organoids, and xenograft tumors (Figs. S2A–G). Together, these data suggest that exogenous l-cysteine uptake is critical for colorectal tumors.

### 3.2 Both cystine and cysteine transporters are upregulated to maintain intracellular cysteine levels in CRC

**Fig. 2:**
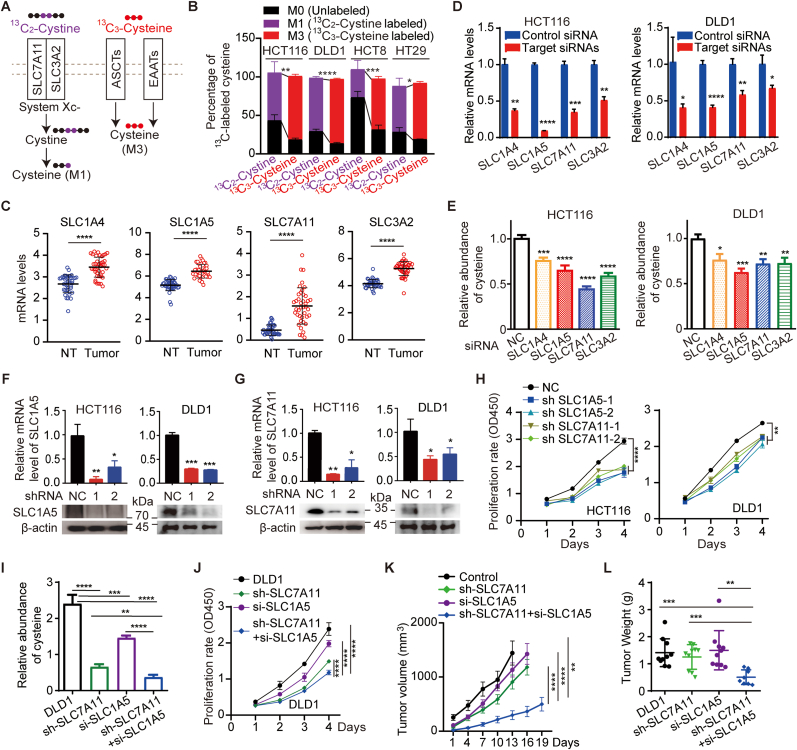
**Cystine/Cysteine transporters are upregulated in CRC cells to promote tumor growth**. (A) Schematic diagram of exogenous ^13^C_2_-cystine or ^13^C_3_-cysteine uptake through various transporters. (B) Colorectal cancer cells import both cystine and cysteine. HCT116 or DLD1 cells was treated by either ^13^C_2_-cystine (100 μM) or ^13^C_3_-cysteine (100 μM). Intracellular cysteine was labeled as ^13^C_1_-cysteine (M1) by ^13^C_2_-cystine treatment, or as ^13^C_3_-cysteine (M3) by ^13^C_3_-cysteine treatment. Both unlabeled and ^13^C-labeled cysteine were presented (n = 6). (C) The transporter genes of both cystine and cysteine were significantly upregulated in CRC compared with paired non-tumor colon tissues in TCGA database (n = 50). SLC7A11, the light chain of cystine/glutamate antiporter system; SLC3A2, the heavy chain of cystine/glutamate antiporter system; SLC1A4, alanine/serine/cysteine/threonine transporter 1 (ASCT1); SLC1A5, alanine/serine/cysteine/threonine transporter 2 (ASCT2). mRNA levels represent FPKM value downloaded from TCGA database. (D and E) Knockdown of SLC1A4, SLC1A5, SLC7A11 or SLC3A2 reduced intracellular cysteine abundance in CRC cells. SLC1A4, SLC1A5, SLC7A11 or SLC3A2 was knocked down by siRNAs mixture in HCT116 or DLD1 cells. The knockdown efficiency was validated by qRT-PCR (n = 3) (D), and intracellular cysteine abundance was measured by UHPLC-QTRAP/MS (n = 6 for DLD1 SLC1A4 knockdown cells; n = 3 for others) (E). (F–H) Knockdown of SLC7A11 or SLC1A5 reduced the proliferation of CRC cells. SLC1A5 or SLC7A11 was knocked down by shRNA in HCT116 or DLD1 cells. The knockdown efficiency of SLC1A5 (F) and SLC7A11 (G) was validated by qRT-PCR (n = 3) and Western blots. The proliferation rates were measured by CCK-8 (n = 3) (H). (I–L) Knockdown of SLC1A5 on top of SLC7A11 knockdown further reduced intracellular cysteine content and significantly impaired CRC tumor growth. SLC1A5 was knockdown by siRNAs in DLD1 cells or DLD1 shRNA-SLC7A11 knockdown cells, and the cells were assayed for abundance of intracellular cysteine by UHPLC-QTRAP/MS (n = 3) (I); cell proliferation by CCK-8 (n = 3) (J); xenograft tumor (n = 10) growth rate (K) and weight (L). Two-way ANOVA was used for statistical analyses of H, J, K. Student t-test was used for others. Data of K is presented as mean ± SEM, and data of others are presented as mean ± SD. *p < 0.05; **p < 0.01; ***p < 0.001; ****p < 0.001; n.s., not significant.

Human plasma contains both free cystine and cysteine in a range of 10∼100 μM, and the concentration of cystine is usually two to three folds higher than that of cysteine [[29], [30], [31], [32]]. We then treated CRC cells with either ^13^C_2_-labeled cystine or ^13^C_3_-labeled cysteine (Fig. 2A). As shown in Fig. 2B, CRC cells imported both cystine and cysteine. Interestingly, it appeared that cysteine was more accessible than cystine for CRC cells (Fig. 2B).

To understand how CRC cells highly import both cystine and cysteine synchronously, we next evaluated the expression of cystine and cysteine transporters in TCGA COAD and READ databases as well as our RNA-seq data (GSE223120). The xCT antiporter consisting of SLC7A11 light chain and SLC3A2 heavy chain is responsible for cystine uptake, and cysteine is majorly transported by ASCTs (SLC1A4, SLC1A5). The expressions of both cystine transporters (SLC7A11, SLC3A2) and cysteine transporters (SLC1A4, SLC1A5) were significantly upregulated in CRC compared with paired non-tumor tissues (Fig. 2C and S3A), but were similar in primary tumors and metastatic tumors (Fig. S3B). Knocking down any one of SLC7A11, SLC3A2, SLC1A4, or SLC1A5 in HCT116 or DLD1 cells reduced their intracellular cysteine abundance (Fig. 2D–G, S3C-E), suggesting that both cystine and cysteine transporters are important for maintaining intracellular cysteine levels in CRC cells. However, the expression of other cystine/cysteine transporters in specific tissues, such as SLC1A1 in neuron [33], SLC3A1 in breast cancer [34], or SLC7A9 in kidney [35], were downregulated or showed no difference between CRC tumors and non-tumor samples (Fig. S3F).

### 3.3 Knockdown of cystine/cysteine transporters impair the growth of CRC

Given the important role of cystine/cysteine for CRC growth, blocking the uptake of cystine or cysteine should affect CRC growth. As expected, knockdown of either SLC1A5 or SLC7A11 reduced the proliferation rates of both HCT116 and DLD1 cells (Fig. 2H). More importantly, knockdown of SLC1A5 with siRNAs in DLD1 SLC7A11 knockdown cells could further reduce intracellular cysteine levels (Fig. 2I), proving that CRC cells maintain their intracellular cysteine levels by importing both exogenous cystine and cysteine simultaneously. Consistently, SLC1A5 and SLC7A11 double knockdown cells exhibited slower growth rates in both in vitro cell culture and in vivo subcutaneous xenograft tumors compared with either SLC1A5 or SLC7A11 single knockdown cells (Fig. 2J–L). These data suggest that both exogenous cystine and cysteine uptake are important for maintaining high cellular cysteine levels in CRC cells, therefore supporting CRC survival and growth.

### 3.4 Cystine/cysteine transporters are upregulated by hypoxia

**Fig. 3:**
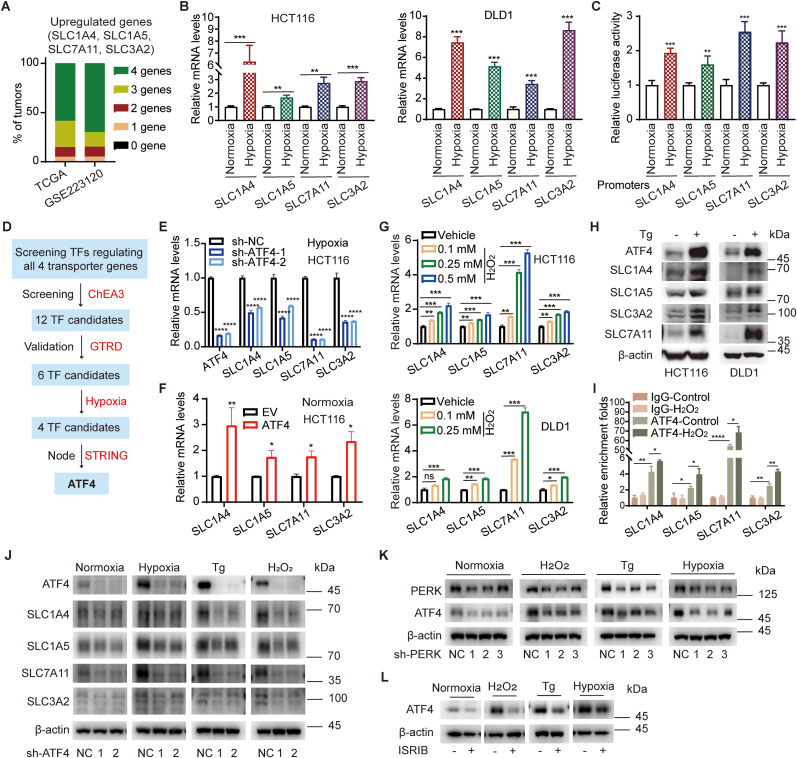
**Hypoxia induces the transcription of cystine/cysteine transporters through ATF4**. (A) SLC1A4, SLC1A5, SLC7A11 and SLC3A2 were upregulated concurrently in CRC tumors. The RNA-seq data of SLC1A4, SLC1A5, SLC7A11 and SLC3A2 in CRC and paired non-tumor tissues from TCGA (n = 50) and GSE223120 (n = 20) were analyzed. The fold change (tumor/non-tumor) >1.1 was considered as upregulation. The percentage of tumors which upregulated 4 genes, 3 genes, 2 genes, or 1 gene were presented. (B and C) Hypoxia induced the expression of SLC1A4, SLC1A5, SLC7A11 and SLC3A2. HCT116 and DLD1 cells were incubated in hypoxia chamber (1.0 % O_2_, 5.0 % CO_2_, 94 % N_2_ 37 °C) or normoxia chamber overnight respectively. qRT-PCR was performed to examine the expression levels of SLC1A4, SLC1A5, SLC7A11 and SLC3A2 (n = 3) (B). Two independent sets of HCT116 cells were transfected with indicated pGL3-promoter constructs and internal control Renilla luciferase construct. 2 days post-transfection, two sets of cells were incubated under hypoxia or normoxia conditions respectively overnight. Luciferase activities were then assayed by dual-luciferase reporter assay system (n = 3) (C). (D) The flowchart to screen transcription factors which are responsible for co-expression of SLC1A4, SLC1A5, SLC7A11 and SLC3A2. ChEA3, ChIP-X Enrichment Analysis Version 3; GTRD, Gene Transcription Regulation Database; Hypoxia, the transcription factors which were reported as hypoxia inducible genes; STRING, functional protein association networks. (E and F) Hypoxia-related ATF4 regulates the expression of SLC1A4, SLC1A5, SLC7A11 and SLC3A2. ATF4 was knocked down by shRNAs in HCT116 cells. Cells were incubated under hypoxia condition overnight and the expression levels of indicated genes were examined by qRT-PCR (n = 3) (E). pCMV-ATF4 or empty vector was transfected into HCT116 cells. 48 h post-transfection, the expression levels of indicated genes were examined by qRT-PCR (n = 3) (F). (G) ROS induced the expression of SLC1A4, SLC1A5, SLC7A11 and SLC3A2. Cells were treated with H_2_O_2_, and the expression levels of indicated genes were examined by qRT-PCR (n = 3). (H) ER stress regulates the expression of SLC1A4, SLC1A5, SLC7A11 and SLC3A2 via ATF4. HCT116 cells were treated with Tg (thapsigargin), and the expression levels of indicated genes were examined by Western blots. (I) Fold enrichment of DNA fragments surrounding putative ATF4 binding sites of indicated gene promoters by ChIP-qPCR with or without H_2_O_2_ (250 μM) treatment (n = 3). (J) Knockdown of ATF4 significantly inhibited the expression of cystine and cysteine transporter genes induced by hypoxia (overnight), H_2_O_2_ (250 μM, 6 h), or Tg (250 nM, 6 h). (K and L) Hypoxia induced ATF4 expression through PERK. ATF4 protein levels in PERK knockdown cells (K) or ISRIB-treated cells (*p*-eIF2α inhibition) (L) were detected. Student t-test was used for statistical analyses. Data are presented as mean ± SD. *p < 0.05; **p < 0.01; ***p < 0.001; ****p < 0.001; n.s., not significant.

These four transporter genes showed a synchronized upregulation trend in colorectal tumors. In 50 paired tumors and adjacent non-tumor tissues from TCGA COREAD database, 27 of 50 (54 %) CRC samples showed upregulation of SLC1A4, SLC1A5, SLC7A11 and SLC3A2 simultaneously (Fig. 3A), and 17 of 50 (34 %) CRC samples showed upregulation of three transporter genes (Fig. 3A). In our GEO data (GSE223120), four genes were simultaneously highly expressed in 14 of 20 tumors (70 %) (Fig. 3A), and more than 80 % tumors overexpressed at least three of four transporter genes overall, suggesting that a common signaling pathway regulates their transcription. Hypoxia is a hallmark of tumors [36]. The expression of SLC7A11, SLC3A2, and SLC1A5 have been reported to relate to hypoxia in different cell types [37,38]. Thus, we verified whether hypoxia was responsible for the upregulation of these four genes in CRC. As shown in Fig. 3B and C, the transcription of SLC7A11, SLC3A2, SLC1A4, and SLC1A5 in colorectal cancer cells were all activated under hypoxia condition.

### 3.5 Hypoxia induces the transcription of cystine/cysteine transporters through ATF4

We next set out to determine how hypoxia induces the expression of these four transporter genes in CRC. We hypothesize that these transporters may have co-transcription factors (TFs), which are responsive to hypoxia. However, knockdown of the classic hypoxia induced factors, either HIF1α (encoded by HIF1A) or HIF2α (encoded by HIF2A), couldn't consistently decrease the expressions of all four transporters under hypoxia (Figs. S4A and B). To identify the common TFs of these four cystine/cysteine transporters, ChEA3 (ChIP-X Enrichment Analysis Version 3) [39] was firstly applied to predict transcription factors co-expression network (Fig. 3D). 12 candidates were identified as TFs for all four transporters, and 6 of them were validated by Gene Transcription Regulation Database (GTRD) (Fig. 3D and S4C). Among 6 TF candidates, ATF4, DDIT3 (CHOP), JUN (AP-1), and BACH2 are related to hypoxia (Fig. S4C) [[40], [41], [42], [43]]. With STRING analysis (https://cn.string-db.org/), ATF4 was identified as the core node for the co-expression of SLC1A4, SLC1A5, SLC7A11, and SLC3A2 (Fig. 3D and S4D). Similar as SLC1A4, SLC1A5, SLC7A11, and SLC3A2, the expression of ATF4 was induced by hypoxia (Fig. S4E). ATF4 was highly expressed in both mRNA levels and protein levels in CRC tumors (Figs. S4F and G). Moreover, CRC patients with high levels of ATF4 showed worse overall survival (Fig. S4H). Knockdown of ATF4 in HCT116 cells reduced the transcription levels of the four transporters under hypoxia condition (Fig. 3E), and overexpression of ATF4 increased their transcription levels under normoxia condition (Fig. 3F). These data suggest that transcription factor ATF4 is involved in regulating the transcription of cystine/cysteine transporters in CRC. Notably, at the protein levels, SLC1A4 and SLC1A5 were highly expressed in most CRC tumors, and SLC7A11 was highly expressed in more than half of tumor samples (Fig. S4G). SLC3A2, surprisingly, was upregulated in CRC only according to lower-molecular-weight bands (Fig. S4G). It is possible that these transporters may also be regulated in protein translation or post-translational processes.

ATF4 is a key coordinator of the integrated stress response (ISR) [44]. Reactive oxygen species (ROS) induced by hypoxia promote endoplasmic reticulum (ER) stress to activate PERK-eIF2α-ATF4 axis in cancer cells [45,46]. We next examined whether the expression of cystine/cysteine transporters was induced by ROS or ER stress. As expected, H_2_O_2_ treatment (ROS) significantly induced the expression of these transporter genes in a dose-dependent manner (Fig. 3G), and thapsigargin (Tg, ER stress inducer) treatment also induced the expression of SLC1A4, SLC1A5, SLC7A11, SLC3A2, as well as ATF4 (Fig. 3H). ChIP-PCR analyses showed that ATF4 bound to promoters of these transporters, which were further enhanced by H_2_O_2_ treatment (Fig. 3I). Moreover, knockdown of ATF4 significantly inhibited the induction of these transporter genes by hypoxia, H_2_O_2_, or Tg (Fig. 3J). These data suggest that the transcriptional activation of cystine/cysteine transporter genes by ATF4 in CRC is mediated by hypoxia-induced ROS and ER stress.

Furthermore, we checked whether hypoxia/ROS/ER stress affected ATF4 through PERK pathway. As shown in Fig. 3K and L, no matter using shRNA to knockdown PERK (encoded by EIF2AK3) or compound ISRIB to inhibit the phosphorylation of eIF2α, the induction of ATF4 by hypoxia, H_2_O_2_, or Tg was significantly inhibited. Interestingly, *p*-eIF2α was highly expressed in CRC tumor samples (Fig. S4G), suggesting that there may be permanent ER stress in CRC tumors. Thus, consistent with other studies, hypoxia-induced ROS and ER stress activate ATF4 expression through PERK pathway in CRC.

### 3.6 Colorectal cancer cells increase the flux from cysteine to GSH to support tumor growth

**Fig. 4:**
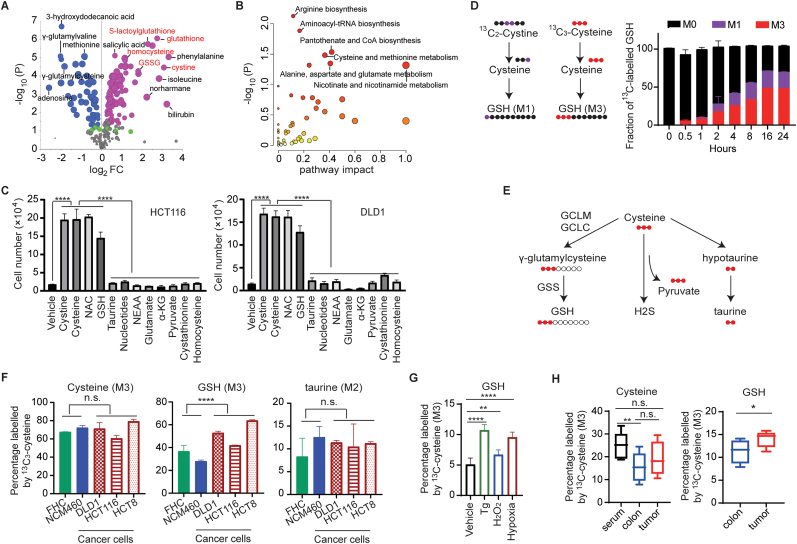
**The flux from cysteine to GSH increases in CRC**. (A) Volcano plots of differential expressed metabolites of HCT116 cells cultured with or without cystine/cysteine. Metabolites in cysteine-GSH pathway are highlighted as red. (B) KEGG pathway analysis of DEMs from HCT116 cells with or without cystine/cysteine. Y axis represents -log_10_ (p value) of pathway analysis. (C) GSH rescued the growth of CRC cells in cystine/cysteine depletion condition. 200 μM of cystine, cysteine, or NAC (N-acetyl cysteine), 400 μM GSH (reduced glutathione) or taurine, nucleotides (1 × EmbyoMax Nucleosides), NEAA (1 × non-essential amino acids), 2 mM glutamate or α-KG (dimethyl α-ketoglutarate), 1 mM sodium pyruvate, 200 μM cystathionine or homocysteine were individually supplemented into cystine/cysteine deficient medium. 48 h later, cell numbers were counted with hemocytometer by Trypan-Blue exclusive assay (n = 3). (D–F) The flux from cysteine to GSH is elevated in CRC cells. Cells were supplied with both 100 μM ^13^C_2_-cystine (GSH labeled as M1) and 100 μM ^13^C_3_-cysteine (GSH labeled as M3) for indicated time points, fraction of ^13^C-labeled GSH was measured (n = 6) (D). Schematic diagram of ^13^C_3_-cysteine flux and related enzymes is shown in (E). Cells were cultured in the presence of 100 μM ^13^C_3_-cysteine for 2 h, and percentages of labeled metabolites in total pool were plotted (n = 6) (F). (G) The flux from cysteine to GSH is elevated by hypoxia induced ER stress. DLD1 cells were treated with Tg (250 nM), H_2_O_2_ (250 μM), or hypoxia overnight, and then incubated with 100 μM ^13^C_3_-cysteine for 30 min. Percentages of labeled GSH in total pool were plotted (n = 8). (H) Spontaneous colon tumors were generated with *CAKP* mice. After infusion with ^13^C_3_-cysteine for 4 h, the serum, colorectal tumors and adjacent non-tumor colon tissues were collected, and percentages of labeled metabolites were plotted (n = 8). Student t-test was used for statistical analyses. Data are presented as mean ± SD. *p < 0.05; **p < 0.01; ***p < 0.001; ****p < 0.001; n.s., not significant. (For interpretation of the references to color in this figure legend, the reader is referred to the Web version of this article.)

Intracellular cysteine is an essential substrate for the synthesis of biomolecules such as proteins and metabolites. To determine how cysteine supports the survival and growth of CRC, we examined the transcriptome and metabolome of HCT116 cells with or without cystine/cysteine. RNA-seq analyses revealed that 539 genes were upregulated and 703 genes were downregulated in cystine/cysteine deprivation condition compared with cystine/cysteine abundance condition (Figs. S5A and B). KEGG enrichment analysis showed that differentially expressed genes (DEGs) were majorly enriched in human disease of cancer, cellular processes such as growth, death, motility, and community, as well as signaling transduction and metabolic pathways (Fig. S5C). With OPLS-DA model, metabolic profilings of HCT116 cells cultured with or without cystine/cysteine were significantly separated (Fig. S5D). Differentially expressed metabolites (DEMs) were involved in GSH metabolism (cystine, homocysteine, glutathione, and glutathione disulfide); amino acids metabolism (methionine, isoleucine, phenylalanine, and γ-glutamylvaline); and nucleotide metabolism (adenosine) (Fig. 4A and B). To assess the pivotal metabolites which mediate the function of cystine/cysteine in CRC, we supplied some metabolites individually into cysteine/cystine depletion culture medium to rescue the growth of CRC cells. As shown in Fig. 4C, N-acetyl cysteine (NAC) and reduced glutathione (GSH), but not other non-essential amino acids (NEAA), nucleotides, glutaminolysis, or glycolysis metabolites, rescued the survival and growth defects caused by cystine/cysteine depletion, suggesting cystine/cysteine supports CRC growth majorly through intracellular GSH synthesis.

We then traced the cysteine-GSH flux in vitro and in vivo. When HCT116 cells were supplied with both ^13^C_3_-cysteine and ^13^C_2_-cystine, as is the case with human serum, cysteine-generated GSH (M3) was much more than cystine-generated GSH (M1) (Fig. 4D), further supporting the previous conclusion that cysteine is more accessible than cystine for CRC cells. Although exogenous uptake was the primary source (about 60–80 %) for intracellular cysteine in both colon cancer cells (DLD1, HCT116, and HCT8) and normal epithelial cells (FHC and NCM460) (Fig. 4E and F), the fractionation of ^13^C_3_-labeled GSH was significantly higher in colon cancer cells than that in normal epithelial cells (Fig. 4F). Cysteine-GSH flux was further enhanced by hypoxia, H_2_O_2_, or Tg treatment (Fig. 4G and S5E). Whereas, ^13^C-labeled taurine had no difference between colon cancer cells and normal epithelial cells (Fig. 4F), and ^13^C-labeled pyruvate or serine was not detectable. These data were further validated with an in vivo model (Fig. 4H). Compared with their paired normal colon tissues, colon tumors from *CAKP* mice generated more GSH from cysteine (Fig. 4H). These data suggest that CRC cells increased their cysteine-GSH flux to support CRC growth. Surprisingly, normal colon epithelial cells showed a similar fraction of intracellular ^13^C-labeled cysteine as colorectal cancer cells in vitro, however, although not significant, the fractionation of ^13^C-labeled cysteine was higher in tumor tissues than normal colon tissues in mice. It might be due to that exogenous cysteine uptake is necessary for normal colon epithelial cells becoming immortal when they are cultured in vitro.

### 3.7 Overexpression of GSS contribute to the increase of cysteine-GSH flux in CRC

**Fig. 5:**
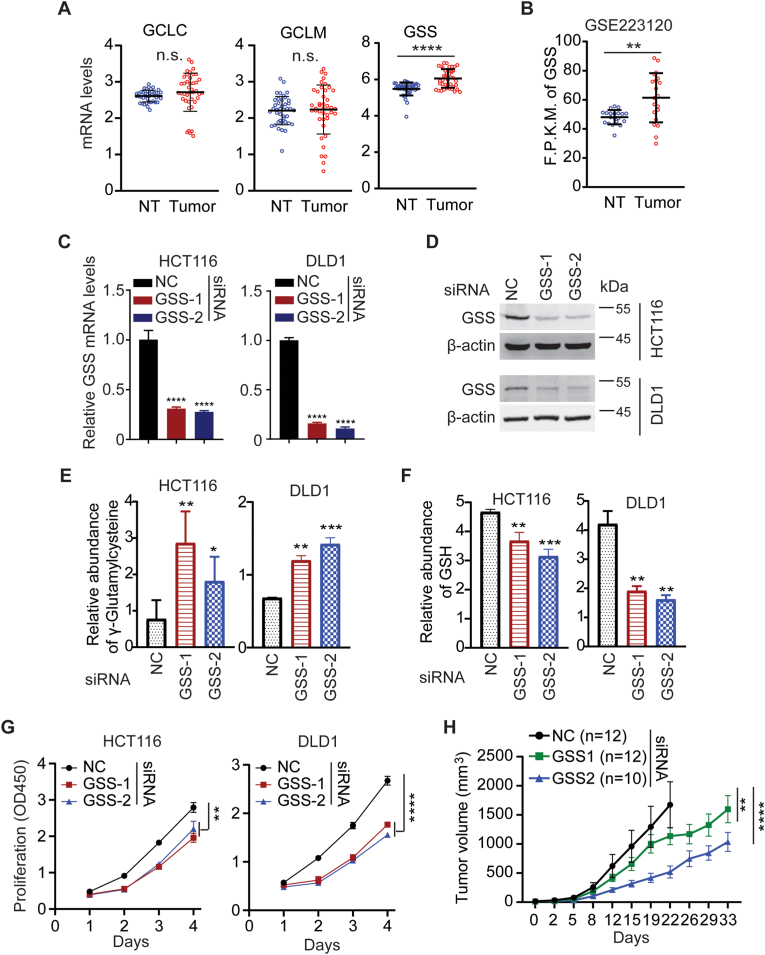
**Overexpression of GSS plays important role in CRC growth**. (A and B) Glutathione synthetase (GSS) was significantly upregulated in CRC samples compared with normal colon tissues. The mRNA levels of indicated genes of paired CRC and normal tissues in TCGA (n = 50, A) and GSE223120 (n = 20, B) datasets were plotted. (C–H) Knockdown of GSS reduced the growth of colorectal cancer cells. HCT116 or DLD1 cells were transfected with siRNAs against GSS or control siRNA. 48 h post-transfection, the cells were assayed for knockdown efficiency by qRT-PCR (n = 3) (C) or Western blots (D); relative abundance of γ-glutamylcysteine (E) or GSH (F) by LC-MS (n = 6); cell proliferation rate by CCK-8 (n = 3) (G); subcutaneous xenograft tumor growth (n = 10) (H). Two-way ANOVA was used for statistical analyses of G, H. Student t-test was used for others. Data of H is presented as mean ± SEM, and data of others are presented as mean ± SD. *p < 0.05; **p < 0.01; ***p < 0.001; ****p < 0.001; n.s., not significant.

The GSH generation from cysteine is catalyzed by glutamate cysteine ligases (GCLM and GCLC) and glutathione synthetase (GSS) (Fig. 4E). Among them, GSS was significantly upregulated in CRC samples compared with normal colon tissues (Fig. 5A and B), which might be responsible for the increase of cysteine-GSH flux in CRC cells. We then knocked down GSS in HCT116 and DLD1 cells with siRNAs (Fig. 5C and D). As expected, knockdown of GSS significantly reduced GSH abundance and accumulated γ-glutamylcysteine (Fig. 5E and F), resulting in impairing the growth of CRC cells and subcutaneous xenograft tumors (Fig. 5G and H).

### 3.8 Scavenging cystine/cysteine represses colorectal tumor growth

**Fig. 6:**
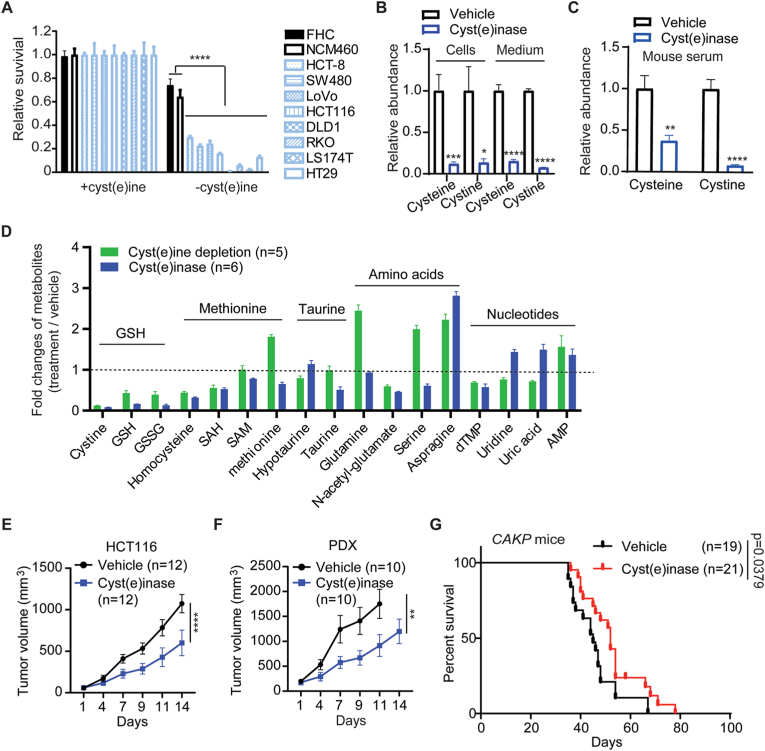
**Scavenging exogenous cystine/cysteine represses CRC growth**. (A) Relative survival of colorectal cancer cells and normal colon epithelial cells under cystine/cysteine depletion condition (n = 3). (B and C) Cyst(e)inase efficiently depletes cystine and cysteine. HCT116 cells were treated with 1 mg/ml cyst(e)inase for 24 h. Intracellular (cells) and exogenous (medium) cystine or cysteine were measured by UHPLC-QTRAP/MS (n = 6) (B). C57BL/6 mice (n = 6) were intraperitoneally injected with cyst(e)inase (50 mg/kg). 24 h later, mice serum was collected to measure cystine or cysteine levels by UHPLC-QTRAP/MS (C). (D) The fold changes of cysteine-related metabolites abundance after HCT116 cells were treated with cystine/cysteine depletion condition or cyst(e)inase for 24 h. (E–G) Cyst(e)inase inhibited the CRC tumor growth. Nude mice were subcutaneous injected with either HCT116 cells (E) or small pieces of patient-derived xenograft tumors (F). Once tumors reached 100–150 mm^3^, mice were divided into two groups to intraperitoneally inject with PBS vehicle or cyst(e)inase (50 mg/kg) thrice a week. *CAKP* mice were administrated with tamoxifen. 40 days post injection, mice were divided into two groups to intraperitoneally inject with PBS vehicle or cyst(e)inase (50 mg/kg) thrice a week. Kaplan-Meier analyses was used to show the survival of mice with or without cyst(e)inase treatment (G). Student t-test was used for statistical analyses. Data are presented as mean ± SD. *p < 0.05; **p < 0.01; ***p < 0.001; ****p < 0.001; n.s., not significant.

As exogenous cystine/cysteine are critical to support CRC growth, depleting cystine/cysteine could be an effective strategy for colorectal cancer therapy. Indeed, cystine/cysteine deprivation in culture medium largely suppressed the growth of CRC cells but was less effective for normal colon epithelial cells (FHC and NCM460) (Fig. 6A). To scavenge exogenous cystine/cysteine in mice, we first attempted to feed mice with cystine/cysteine deficient diet (CDD). However, the serum cystine/cysteine levels were similar in control mice and CDD-feeding mice (Fig. S6A), suggesting that CDD could not achieve the cystine/cysteine depletion effectively. An engineered mutant cystathionine γ-lyase so called “cyst(e)inase” has been reported to deplete cystine/cysteine pool in mice robustly [47]. We then generated cyst(e)inase as described by Cramer et al. [47]. Cyst(e)inase efficiently scavenged intracellular and extracellular cystine and cysteine in CRC cells (Fig. 6B), as well as in mouse serum (Fig. 6C). Cyst(e)inase treatment showed similar metabolic changes as cystine/cysteine depletion, especially regarding the metabolites in GSH pathway (Fig. 6D). Importantly, cyst(e)inase significantly inhibited the growth of subcutaneous CRC tumors generated by either HCT116 cells (CDX model) or a patient-derived xenograft (PDX model) without reducing mice body weight (Fig. 6E and F, and S6B). Furthermore, cyst(e)inase increased survival time of *CAKP* mice harboring colon tumors (Fig. 6G). These data suggest that depletion of cystine/cysteine by cyst(e)inase could be an effective approach to treat CRC.

### 3.9 Depletion of cystine/cysteine induces autophagy through mTOR-ULK pathway

**Fig. 7:**
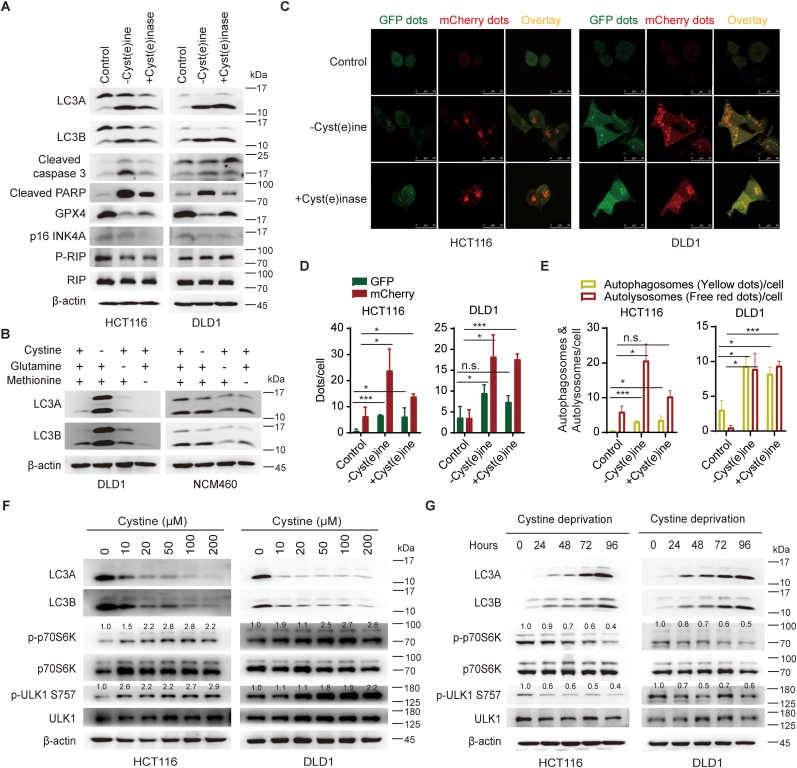
**Depletion of cystine/cysteine induces autophagy of CRC cells through mTOR-ULK pathway**. (A) Depletion of cystine/cysteine affects cellular autophagy, apoptosis or ferroptosis. Cells were cultured under cystine/cysteine depletion condition (-cyst(e)ine) or treated with cyst(e)inase (+cyst(e)inase) for 48 h. Cell lysates were collected and cell death markers were detected by Western blot assays. (B) Depletion of cystine/cysteine, but not glutamine or methionine, significantly induced autophagy in CRC cells but not in normal epithelial cells. (C–E) Cystine/cysteine depletion significantly induces autophagy in CRC cells. mCherry-GFP-LC3-expressing HCT116 and DLD1 cells were cultured under cystine/cysteine depletion condition or treated with cyst(e)inase for 24 h. Representative images of fluorescent LC3 puncta were show in (C). Mean number of GFP and mcherry dots per cell (n = 3) (D); and mean number of autophagosomes (yellow dots in merged images) and autolysosomes (red dots in merged images) per cell were counted (n = 3) (E). (F and G) Cystine/cysteine depletion induces autophagy of CRC cells through mTOR-ULK1 pathway. The dose-dependence (F) and time-dependence (G) of autophagy (the expression of LC3A and LC3B), mTOR activity (p-p70S6K) and ULK activity (*p*-ULK) upon cystine/cysteine depletion were assayed by Western blots. Student t-test was used for statistical analyses. Data are presented as mean ± SD. *p < 0.05; **p < 0.01; ***p < 0.001; ****p < 0.001; n.s., not significant. (For interpretation of the references to color in this figure legend, the reader is referred to the Web version of this article.)

We next set out to elucidate the molecular mechanism by which cystine/cysteine depletion inhibits the growth of CRC. Depletion of cystine/cysteine has been shown to elicit autophagic responses or ferroptosis in cancer cells [[47], [48], [49]]. In CRC cells, upon depleting cystine/cysteine or cyst(e)inase treatment, the expression of LC3A/B, cleaved PARP, and cleaved caspase 3 were upregulated, and GPX4 expression was downregulated (Fig. 7A). But depletion of cystine/cysteine had no effect on the expression of p16 INK4A and p-RIP (Fig. 7A). Interestingly, either ferroptosis inhibitor ferrostatin-1 (Ferr-1) or apoptosis inhibitor Z-VAD-FMK (ZVAD) couldn't prevent cell death caused by cystine/cysteine deprivation (Fig. S7A). However, autophagy inhibitors (3-MA or CQ) could further suppress the growth of CRC cells on top of cystine/cysteine depletion condition (Figs. S7B and C), suggesting that autophagy plays an essential role in CRC cell viability upon cystine/cysteine depletion. Depletion of cystine/cysteine, but not other related amino acids (methionine or glutamine), specifically induced autophagy in CRC cells, but had no effect on normal colon epithelial cells (Fig. 7B). Autophagy induced by cystine/cysteine depletion was further validated with mCherry-GFP-LC3 system (Fig. 7C–E). Moreover, cystine/cysteine depletion induced autophagy in a dose- and a time-dependent manner (Fig. 7F and G). Similar as previous study [47], cystine/cysteine depletion suppressed mTOR activity (p-p70S6K) and *p*-ULK to induce autophagy in CRC cells (Fig. 7F and G).

## 4 Discussion

Cysteine enrichment is a key signature of CRC metabolism. Although DEMs in CRC from different studies varied a lot [14], cysteine was repeatedly found to be enriched in CRC in different cohorts with various platforms. Qiu et al. identified that cysteine was highly enriched in CRC with 376 surgical specimens from four different hospitals by gas chromatography time-of-flight mass spectrometry (GC-TOF MS) [16]. Satoh et al. found the enrichment of cysteine in CRC by capillary electrophoresis time-of-flight mass spectrometry (CE-TOF MS) [13]. Tian et al. also detected that cysteine was enriched in CRC by ^1^H high resolution magic-angle spinning nuclear magnetic resonance (HRMAS NMR) [19]. Glutathione and GSSG are much easier to be detected in LC-MS platform than that in GC-MS platform. Williams et al. found that glutathione was also accumulated in CRC with LC-MS platform [18], suggesting that cysteine-glutathione pathway is abnormally altered in almost all CRC cohorts. Moreover, cysteine is enriched in both colorectal adenoma and adenocarcinoma [13], and maintains high levels in all tumors at TNM stages from I to IV [13,14]. Therefore, cysteine plays an important role in the initiation and progression of CRC. This phenomenon strongly suggests us to investigate the mechanisms of cysteine metabolic reprogramming in CRC.

With in vitro and in vivo stable isotope tracing experiments, we clarify that both cystine and cysteine uptake are essential for cysteine enrichment in CRC. Cystine uptake by xCT is considered as a predominant source for most cancer cells [24]. However, chronic lymphocytic leukemia cells transport cysteine but not cystine [26]. In pancreatic ductal adenocarcinoma (PDAC) cells, cysteine uptake could compensate for the intracellular cysteine pool when SLC7A11 is knocked out [27,28]. Unlike those cancer types, we provide compelling evidence to illustrate that cysteine enrichment in CRC depends on both cystine and cysteine uptake simultaneously. Firstly, majority of intracellular cysteine in CRC is from exogenous intake of both cystine and cysteine (Fig. 2). Secondly, over 80 % CRC tumors highly express both cystine and cysteine transporters synchronously (Fig. 2, Fig. 3). Thirdly, knockdown of these transporters individually decreases intracellular cysteine content, and double-knockdown of both cysteine and cystine transporters could achieve cysteine reduction more significantly in CRC cells (Fig. 2). Interestingly, it has been reported that CRC cell lines are more resistant to ferroptosis induced by erastin (SLC7A11 inhibitor) than other cancer cells [50]. It is probably due to that just blocking SLC7A11 cannot eliminate intracellular cysteine efficiently, whereas blocking both cysteine and cystine import might induce ferroptosis more effectively in colorectal cancer cells.

We demonstrate that both cystine and cysteine are crucial for CRC tumor growth. Firstly, either cysteine or cystine is sufficient to support the growth of cancer cells, organoids, and tumors (Fig. S2). Secondly, depletion of exogenous cysteine/cystine inhibits the growth of cancer cells and tumors (Fig. 6). Thirdly, both cysteine and cystine contribute to intracellular cysteine and GSH pools in CRC cells (Fig. 2, Fig. 4). Lastly, knockdown of cystine or cysteine transporters inhibit the growth of CRC cells and tumors (Fig. 2). Those results have clearly clarified the importance of cysteine in CRC growth, although SLC1A4 and SLC1A5 are neutral amino-acid transporters for not only cysteine but also alanine, serine, threonine or glutamine. Scalise et al. report that cysteine is not a major substrate but a modulator of SLC1A5 [51]. We have now shown that knockdown of SLC1A5 by either siRNA or shRNA reduced intracellular cysteine in CRC cells, however, we have no sufficient evidence to clarify whether SLC1A5 affects intracellular cysteine directly or indirectly. Moreover, whether there are other cysteine transporters involved in CRC growth might be interesting to investigate in the future.

ATF4 is a stress-induced transcription factor which is frequently upregulated in cancer cells [52]. Tumor microenvironment could activate eIF2α-ATF4 axis through several ways such as hypoxia-induced ROS causing ER stress to activate PERK, or amino acid limitation to activate GCN2 [[53], [54], [55], [56]]. Our data demonstrate that hypoxia-ROS-ER stress-ATF4 axis regulates the expression of both cysteine and cystine transporters simultaneously (Fig. 3). Interestingly, Wu et al. show that cysteine affects mTOR activity through the GCN2-ATF4-SESN2 pathway [22]. These data suggest a dual role for ATF4 in cysteine metabolism. PERK-ATF4 axis firstly responds to hypoxia to increase cystine/cysteine uptake, then cysteine generates GSH for ROS homeostasis and regulates mTOR activity through GCN2-ATF4-SESN2 for protein synthesis, thereby promoting CRC growth.

ROS plays an important role in tumorigenesis, as a low level of ROS can promote cancer, while excessive ROS can induce cancer cell apoptosis [53]. However, the underlying mechanism by which cancer cells maintain ROS at a tumorigenic level to facilitate tumor growth remains unclear. Here, we propose a feedback mechanism to maintain ROS balance in colorectal cancer cells. The high level of ROS caused by tumor microenvironments such as hypoxia increases the uptake of exogenous cystine/cysteine by activating ATF4 axis. Then, overexpression of GSS accelerates the flux from cysteine to GSH, thereby subsequentially eliminating excessive ROS and maintaining ROS below cytotoxic levels to promote CRC progression. Since cysteine is also enriched in other tumors, including breast cancer, esophageal cancer, and prostate cancer by metabolome analyses [[57], [58], [59]], and GCLC, GCLM, or GSS are also upregulated in several cancer types (http://gepia2.cancer-pku.cn/), this feedback mechanism might be generalized to different cancer types. Similar to this theory, Kelly et al. show nutrition limitation promotes cysteine to GSH flux to counter nutrition limitation-induced ROS in the eukaryote D. discoideum [60].

Cystine/cysteine depletion is a potentially effective strategy for colorectal cancer treatment. To achieve cystine/cysteine depletion, cystine/cysteine deficient diets, SLC7A11 inhibitors, and an engineered recombinant cyst(e)inase have been applied in mouse models to treat tumors [22,24,47]. The cystine/cysteine deficient diets could not consistently decrease the cystine/cysteine level in mouse serum with our long-term feeding experiment, suggesting that cystine/cysteine balance can be compensated by endogenous cystine/cysteine synthesis. SLC7A11 inhibitors have not achieved therapeutic benefit for cancer patients yet in several clinical trials, which might be due to that cysteine import compensates intracellular cysteine levels after inhibiting SLC7A11 [25,27,28]. The recombinant cyst(e)inase, which robustly eliminates cystine/cysteine in mouse serum, shows great potential for therapeutic treatment of CRC (Fig. 6). Therefore, developing more effective engineered enzymes which have lower K_m_, higher V_max_, or longer half-life for cystine/cysteine depletion will push forward cystine/cysteine depletion as a treatment strategy for cancer patients. Moreover, in CRC cells, cystine/cysteine depletion triggers autophagy via the mTOR/ULK axis, indicating that combining cystine/cysteine depletion with mTOR or autophagy inhibitors may be a more successful treatment strategy for colorectal cancers. We are pursuing this direction and hope to achieve more successful application of cysteine metabolism in colorectal cancer in the future.

## 5 Conclusions

Our study demonstrated an unrecognized mechanism that both cystine and cysteine uptake are crucial for maintaining high intracellular cysteine levels in CRC, therefore supporting CRC growth. Hypoxia-induced ROS and ER stress are responsible for upregulating of both cystine and cysteine transporters in CRC through transcription factor ATF4. Thereby, depletion of cystine/cysteine can be an effective approach to treat CRC patients.

## Ethics approval and consent to participate

This study was approved by the Ethics Committee at Renji Hospital, Shanghai Jiao Tong University School of Medicine (Shanghai, China). Informed consents were obtained from all participants. Animal experiments were approved by the Animal Ethics Committee at Shanghai Cancer Institute, Renji Hospital, Shanghai Jiao Tong University School of Medicine.

## Consent for publication

All the authors consent for publication.

## Funding

This work was supported by National Natural Science Foundation of China (82372615, 82073044, 81772503 to Y. Hao; 32001046 to Z. Lin, 32001047 to T. Wang); Shanghai Municipal Commission of Health Departments (202240172 to Y. Hao; 20194Y0331 to L. Sun; 20224Y0221 to G. Cui). This work was also supported by the Program for Professor of Special Appointment (Eastern Scholar) at 10.13039/501100013285Shanghai Institutions of Higher Learning (TP2017027, GZ2022007); the Program of 10.13039/501100012247Shanghai Academic/Technology Research Leader (19XD1423500); the Youth Science and Technology Talents in Shanghai Sail Plan of China (19YF1445500 to Z. Lin); and State Key Laboratory of Oncogenes and Related Gene (ZZ-94-2309, ZZ-RCPY-23-26, ZZ-RCPY-24-01 to Y. Hao; SB19-08 to Z. Lin; SB22-16 to G. Cui).

## CRediT authorship contribution statement

**Zhang Lin:** Writing – review & editing, Writing – original draft, Visualization, Validation, Methodology, Funding acquisition, Formal analysis. **Shiyi Yang:** Writing – review & editing, Writing – original draft, Visualization, Validation, Methodology, Formal analysis. **Qianqian Qiu:** Writing – review & editing, Writing – original draft, Visualization, Validation, Formal analysis. **Gaoping Cui:** Validation, Funding acquisition. **Yanhua Zhang:** Software, Resources. **Meilian Yao:** Software. **Xiangyu Li:** Resources. **Chengkun Chen:** Resources. **Jun Gu:** Project administration. **Ting Wang:** Resources. **Peng Yin:** Resources. **Longci Sun:** Resources. **Yujun Hao:** Writing – review & editing, Writing – original draft, Visualization, Validation, Supervision, Project administration, Funding acquisition, Conceptualization.

## Declaration of competing interest

The authors declare no conflict of interest.

## Data Availability

Data will be made available on request.
