# Supplementary material for: Hypoxia-induced cysteine metabolism reprogramming is crucial for the tumorigenesis of colorectal cancer
Source: Redox Biol. 2024 Jul 26;75:103286. doi: 10.1016/j.redox.2024.103286 (PMC11340627; doi:10.1016/j.redox.2024.103286)
Supplement: Multimedia component 1 [file mmc1.docx]

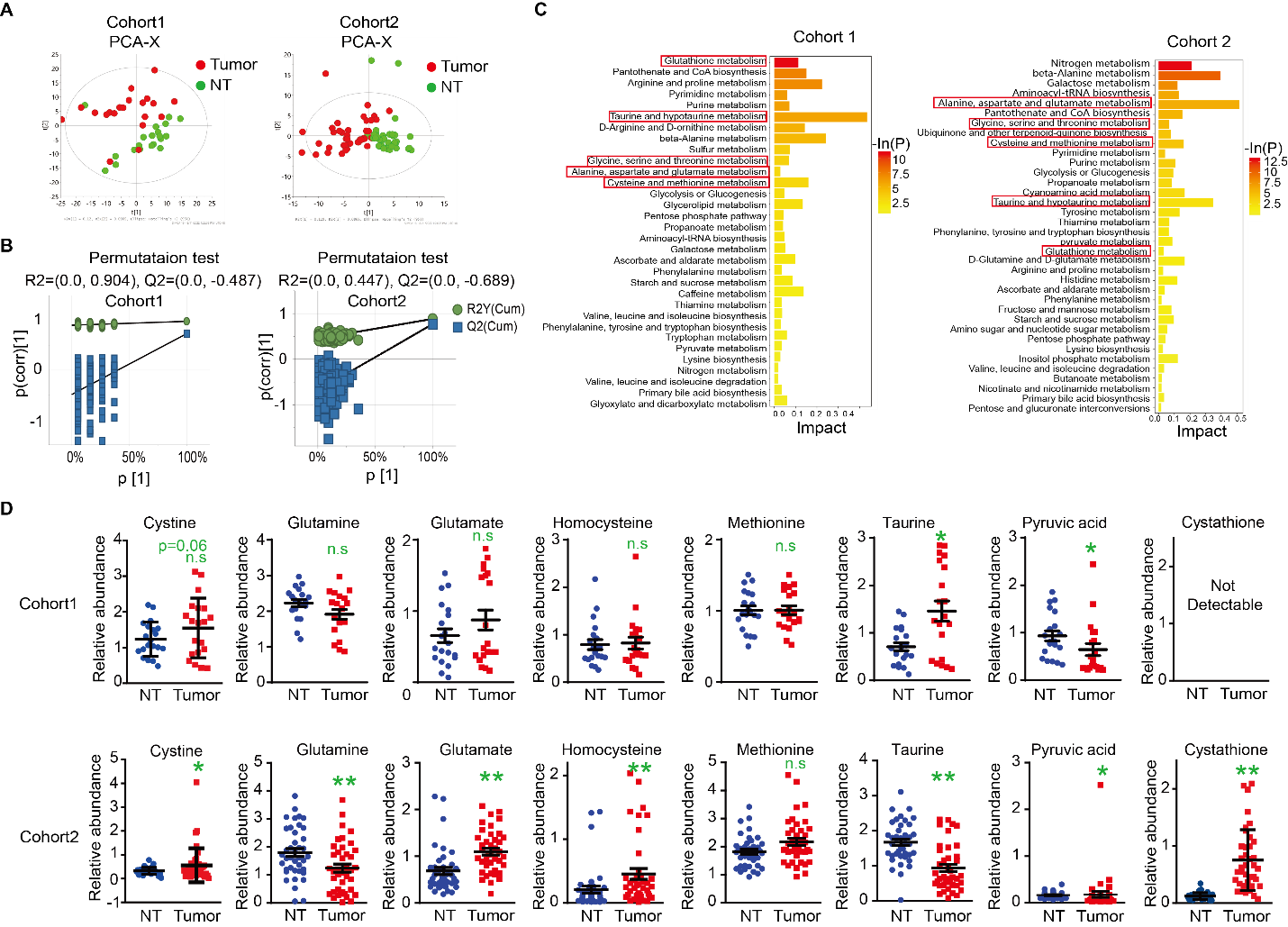


**Fig. S1** **Differential expressed metabolites and metabolic pathways between colorectal cancer tissues and adjacent non-tumor tissues.** (A) PCA scores based on metabolites profiling of tumor samples and adjacent non-tumor samples of two independent cohorts collected from CRC patients (cohort 1, n=20; cohort 2, n=40). (B) Permutation tests of OPLS-DA models. The intercept of R2 and Q2 was a measure of the overfit. A 100-time permutation test was performed to validate the OPLS-DA models. All the R2 and Q2 in the permutation test are lower than the original ones and the intercept for the Q2 to the Y axe were below zero (Q2 intercept (0, -0.487) or (0, -0.689) for cohort1 or cohort 2 respectively), indicating the validity of the OPLS-DA models. (C) KEGG pathway analysis of DEMs from cohort 1 or cohort 2. Red boxes indicate cysteine-related metabolic pathways. (D) The relative abundance of cysteine-related metabolites in cohort 1 and cohort 2.

Student t-test was used for statistical analyses. Data are presented as mean ± SD. *p <0.05; ** p < 0.01; n.s., not significant.


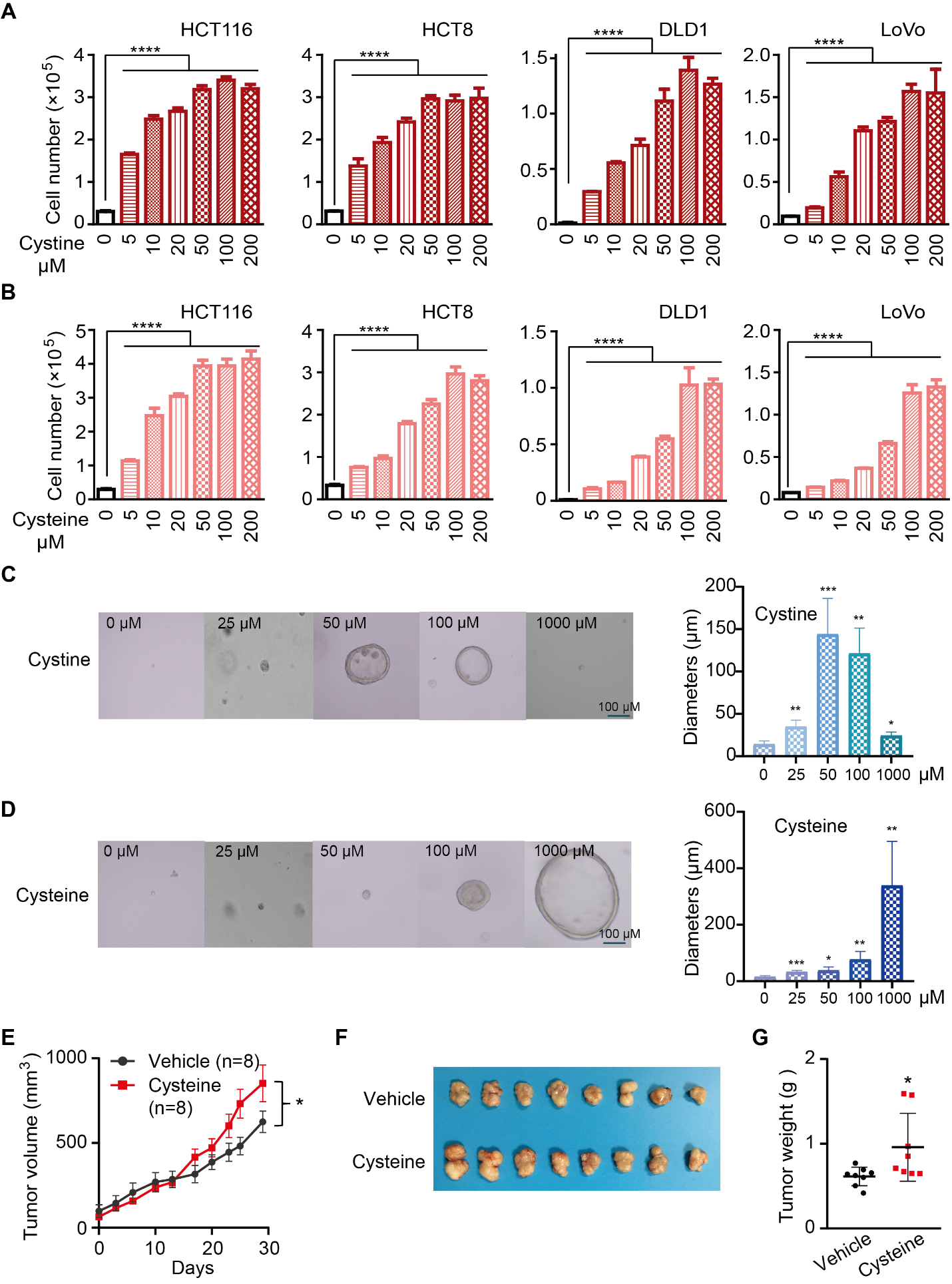


**Fig. S2** **Cystine/Cysteine support the survival and growth of CRC.** (A and B) Cystine (A) or cysteine (B) promotes the proliferation of CRC cells in a dose-dependent manner (n=3). (C and D) Cystine (C) or cysteine (D) supports the growth of organoids generated from colon tumors of *CAPK* mice (n=6 for each group). (E-G) Cysteine promotes the growth of subcutaneous xenograft tumors. HCT116 cells were injected into the flanks of nude mice. When tumor volumes reached 100-150 mm^3^, mice were randomly assigned into two groups to intraperitoneally inject vehicle or cysteine (50 mg/kg) respectively. The tumor growth curve (E), the image of xenograft tumors (F), and tumor weight (G) were presented.

Statistical analyses, two-way ANOVA was used for E, and student's t-test was used for others. Data are presented as mean ± SD of three independent experiments. * p < 0.05; ** p < 0.01.


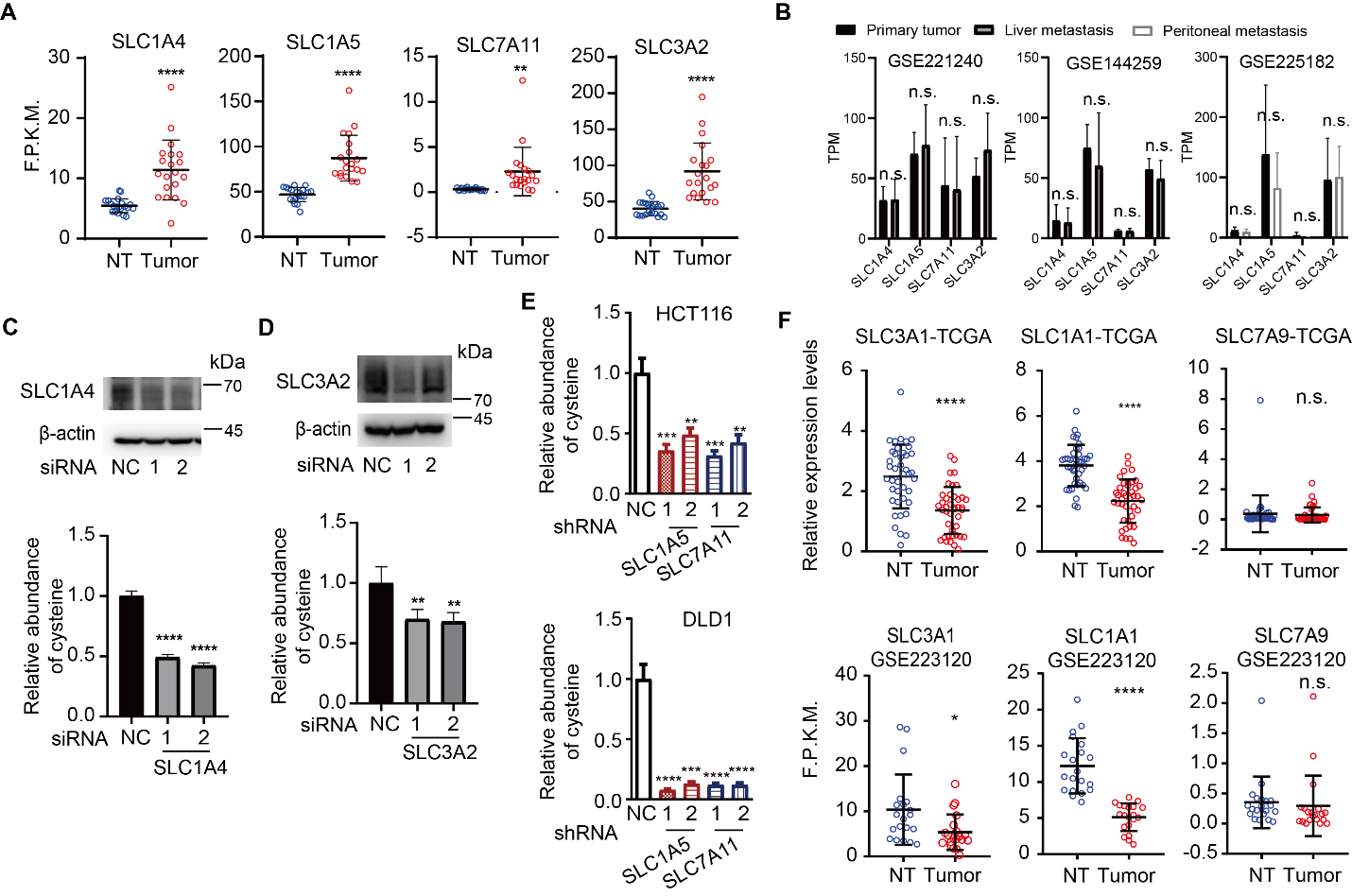


**Fig. S3** **Cystine/cysteine transporters are upregulated in CRC.** (A) The mRNA levels of cystine transporters (SLC7A11, SLC3A2) and cysteine transporters (SLC1A4, SLC1A5) were significantly upregulated in CRC compared with paired non-tumor colon tissues in GEO dataset (GSE223120). (B) The expression levels of SLC1A4, SLC1A5, SLC7A11, and SLC3A2 in CRC primary tumors and metastatic tumors. (C and D) Relative cysteine abundance was reduced by knockdown of SLC1A4 (C) or SLC3A2 (D) with two independent siRNA sequence in HCT116 cells. (E) shRNA-mediated knockdown of either SLC1A5 or SLC7A11 reduced cellular cysteine levels in HCT116 and DLD1 cells. (F) The expression levels of transporter genes for cystine (SLC7A9, SLC3A1) or cysteine (SLC1A1) were not altered or downregulated in CRC compared with paired non-tumor tissues.

Student t-test was used for statistical analyses. Data are presented as mean ± SD. *p <0.05; ** p < 0.01; *** p < 0.001; **** p < 0.001; n.s., not significant.


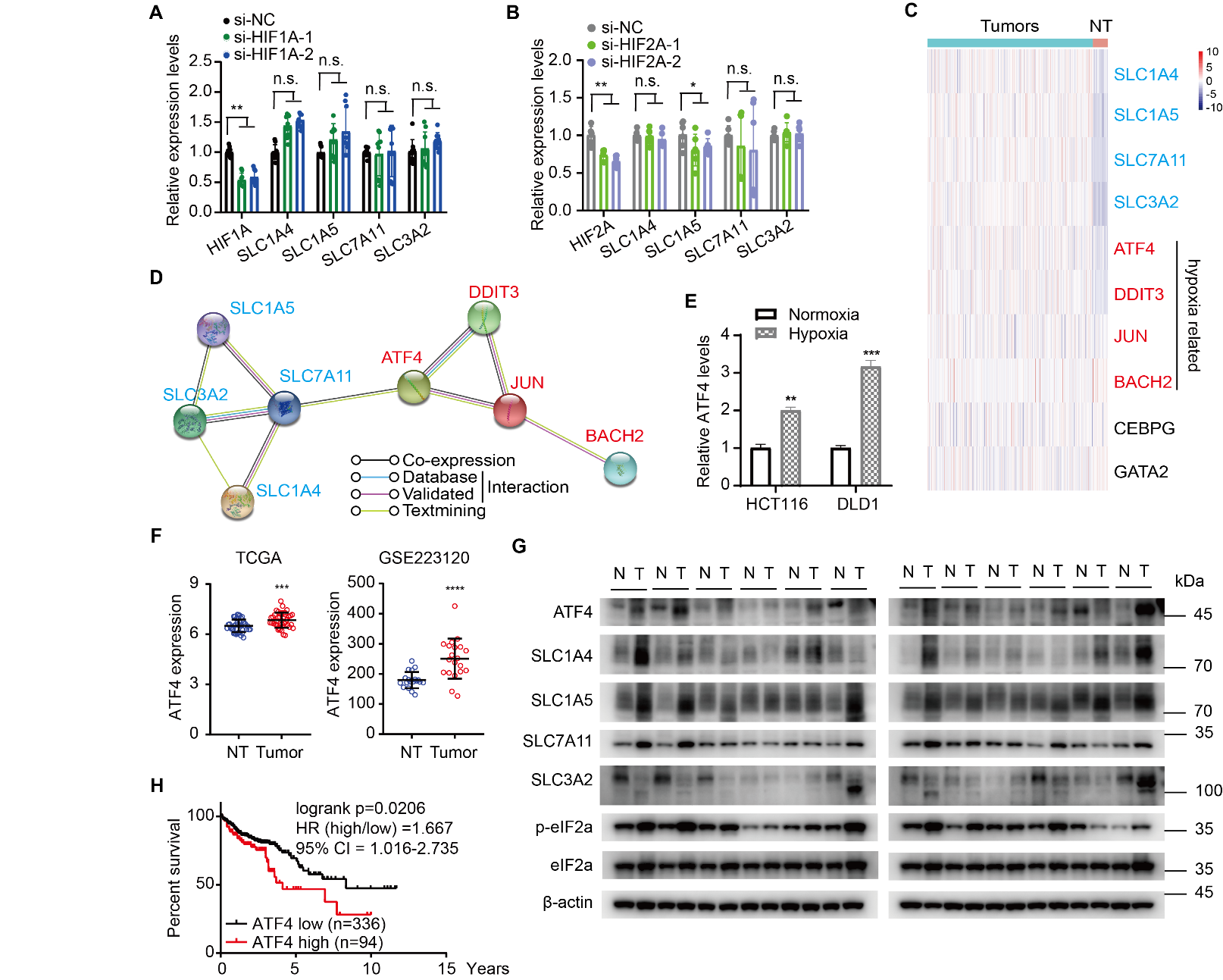


**Fig. S4** **Hypoxia regulates the expression of cystine/cysteine transporters in CRC by ATF4.** (A and B) Cystine/cysteine transporter genes were not consistently regulated by HIF1A (A) or HIF2A (B) in CRC. HCT116 cells were transfected with indicated siRNAs. After 48 hours, cells were treated with hypoxia condition overnight, and RNAs were extracted for qPCR (n=3). (C) The expression of SLC1A4, SLC1A5, SLC7A11, SLC3A2 and their common transcription factors predicted by ChEA3 and GTRD in TCGA database. (D) ATF4 is identified as the core node for co-expression of SLC1A4, SLC1A5, SLC7A11, and SLC3A2 using STRING analysis. (E) Hypoxia induced the expression of ATF4 in HCT116 and DLD1 cells by qRT-PCR (n=3). (F and G) ATF4 is upregulated in CRC. The mRNA levels of ATF4 of paired CRC and normal tissues in TCGA (n=50) and GSE223120 (n=20) datasets were plotted (F). Colorectal tumors (T) and adjacent non-tumor tissues (N) were collected and indicated protein levels were detected by Western blots (G). (H) High levels of ATF4 are associated with worse survival of CRC patients. The overall survival of patients expressing high levels versus low levels of ATF4 were calculated using survival and survminer package of R® ([https://CRAN.R-project.org/package=survminer](https://cran.r-project.org/package=survminer)) based on the Kaplan-Meier method.

Student t-test was used for statistical analyses. Data are presented as mean ± SD. *p <0.05; ** p < 0.01; *** p < 0.001; **** p < 0.001; n.s., not significant.


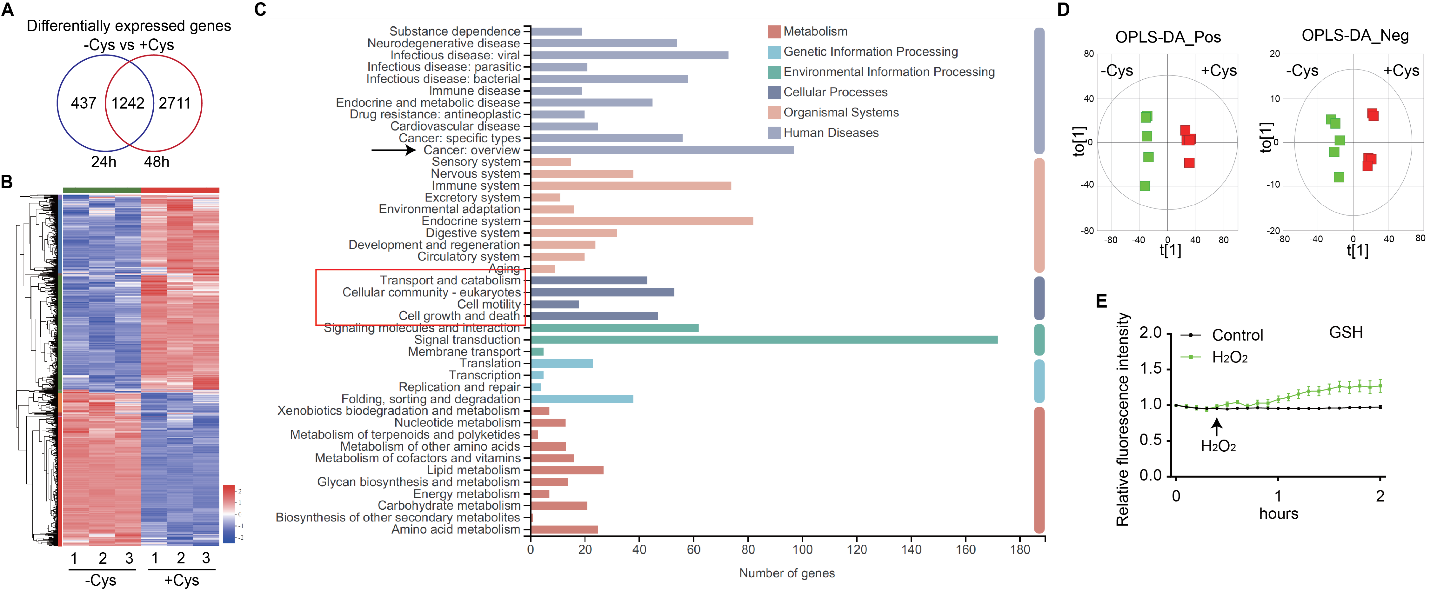


**Fig. S5** **Cystine/cysteine depletion impairs glutathione synthesis to suppress CRC growth.** (A) Venn diagram of the differentially expressed genes (DEGs) of HCT116 cells cultured with or without cystine/cysteine for 24 hours or 48 hours. (B) Heat map of RNAseq transcriptome for 1242 overlapping DEGs. (C) KEGG pathway analysis of 1242 DEGs. The arrowhead indicates that most of DEGs were enriched in human disease of cancer. The red box indicates that most of DEGs were enriched in cellular processes of growth, death, motility, community, and transport. (D) OPLS-DA score plots for metabolites profiling of HCT116 cells cultured with or without cystine/cysteine for 24 hours by LC-TOF-MS. (E) H_2_O_2_ treatment induces GSH generation. GSH fluorescence of 7 independent views were measured with BCC probe (kindly gift from Dr. Peng Yin) every 6 minutes.

Student t-test was used for statistical analyses. Data are presented as mean ± SD. *p <0.05; ** p < 0.01; *** p < 0.001; **** p < 0.001; n.s., not significant.


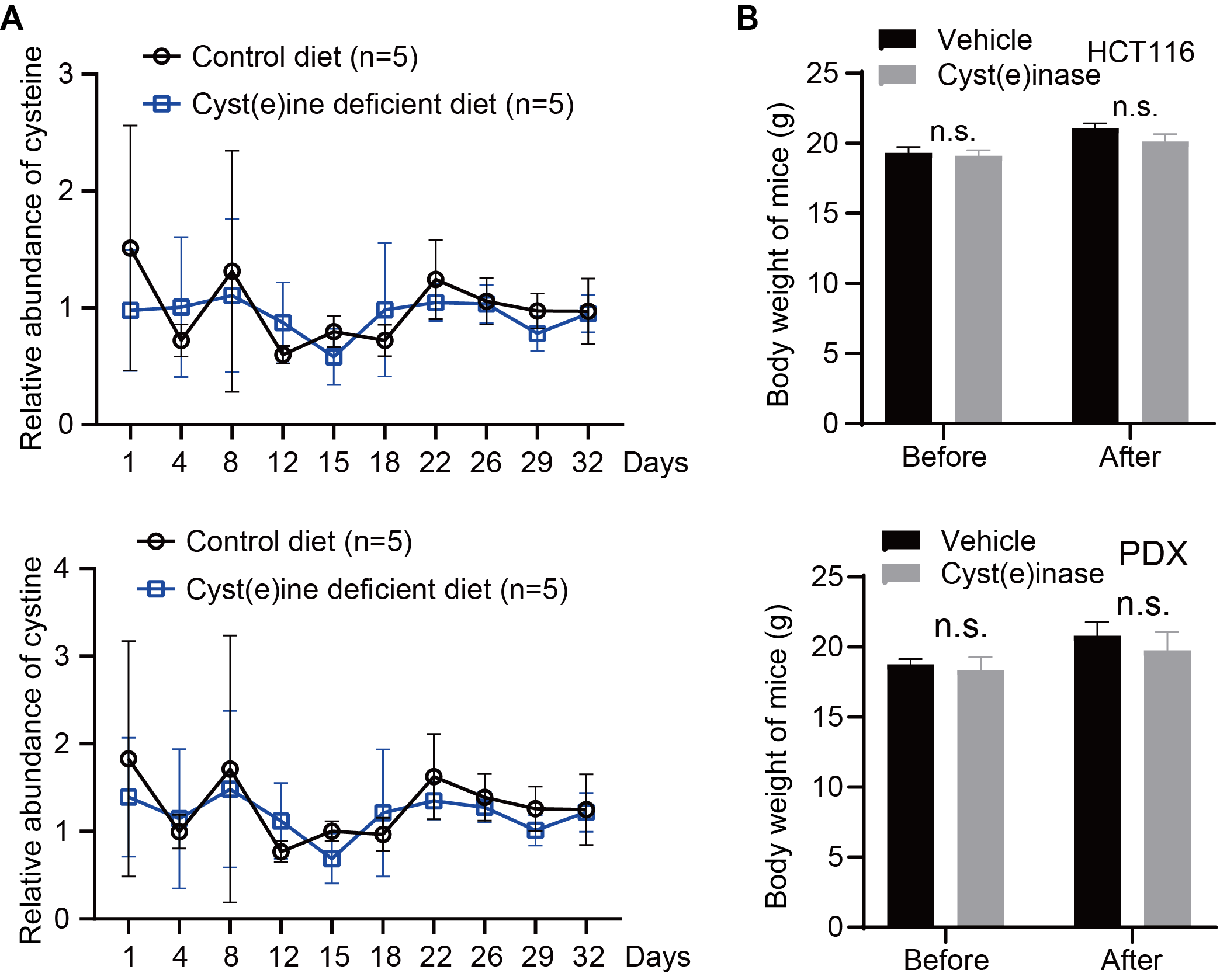


**Fig. S6 Scavenging exogenous cystine/cysteine have no impact on mice weight.** (A) Cyst(e)ine (both cystine and cysteine) Deficient Diet did not consistently reduce serum cystine/cysteine content in C57BL/6 mice. (B) The body weight of mice before and after treated with cyst(e)inase.

Student t-test was used for statistical analyses. Data are presented as mean ± SD, n.s., not significant.


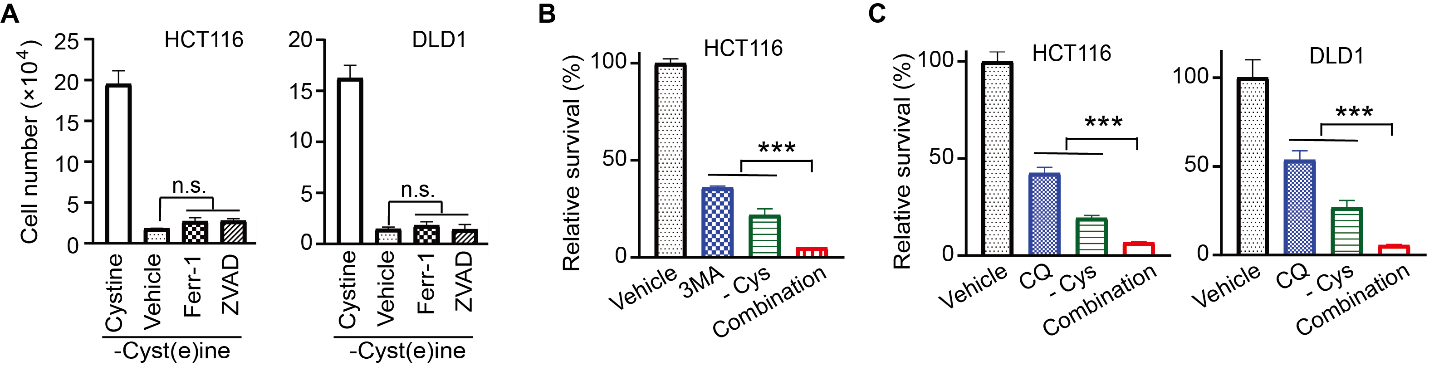


**Fig. S7 Depletion of cystine/cysteine induces autophagy in CRC cells.** (A) The cell death caused by cystine/cysteine depletion cannot be prevented by inhibiting apoptosis or ferroptosis. Cells were cultured under cystine/cysteine depletion condition supplemented with 2 μM ferroptosis inhibitor ferrostatin-1 (Ferr-1) or 5 μM apoptosis inhibitor Z-VAD-FMK (ZVAD). 48 hours later, cell numbers were counted with hemocytometer by Trypan-Blue exclusive assay. (B and C) Autophagy inhibitors 3-MA (B) or CQ (C) further inhibit the growth of CRC cells under cystine/cysteine depletion condition.

Student t-test was used for statistical analyses. Data are presented as mean ± SD. *** p < 0.001;; n.s., not significant.


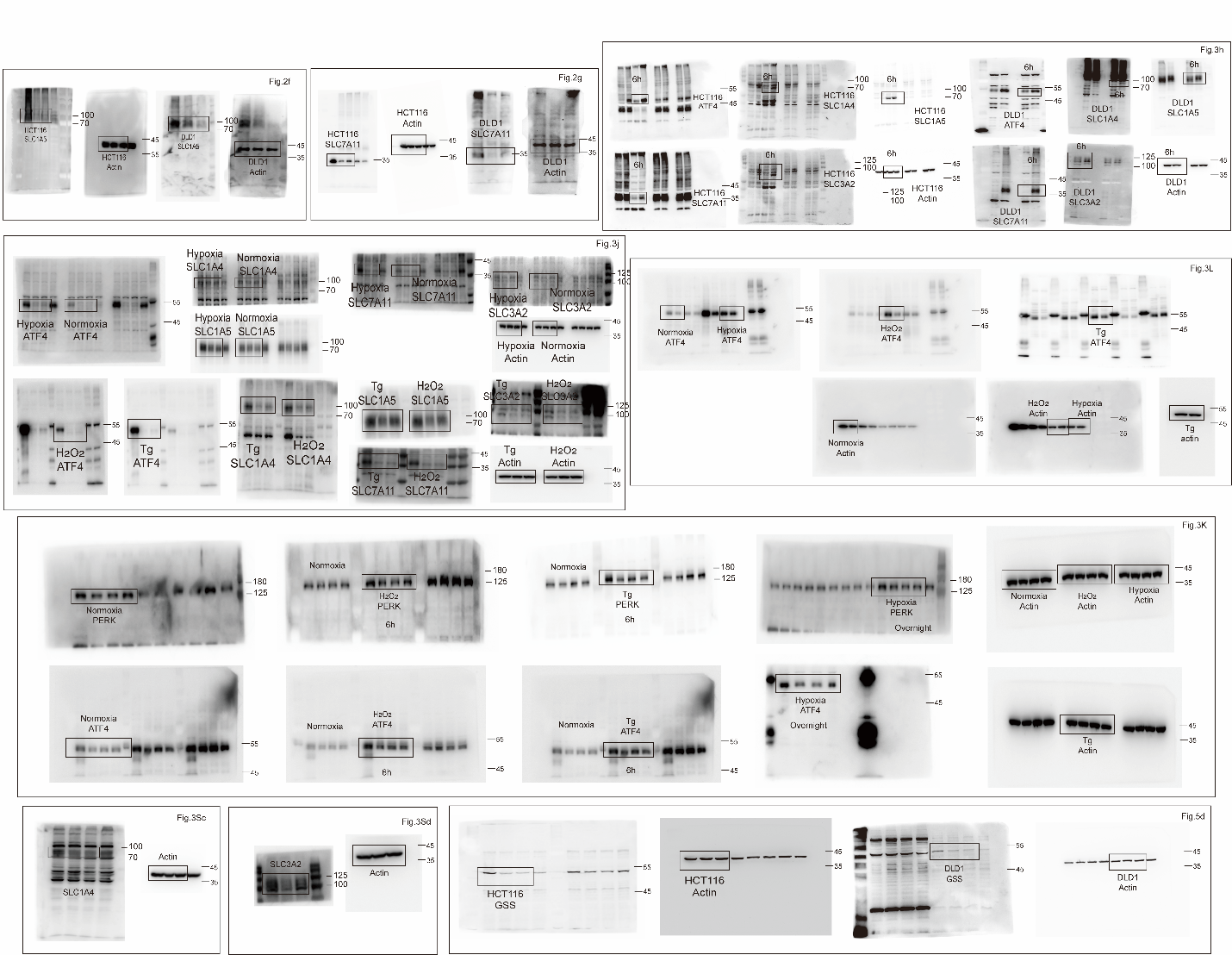


**Fig. S8-1 Original Western blots.**


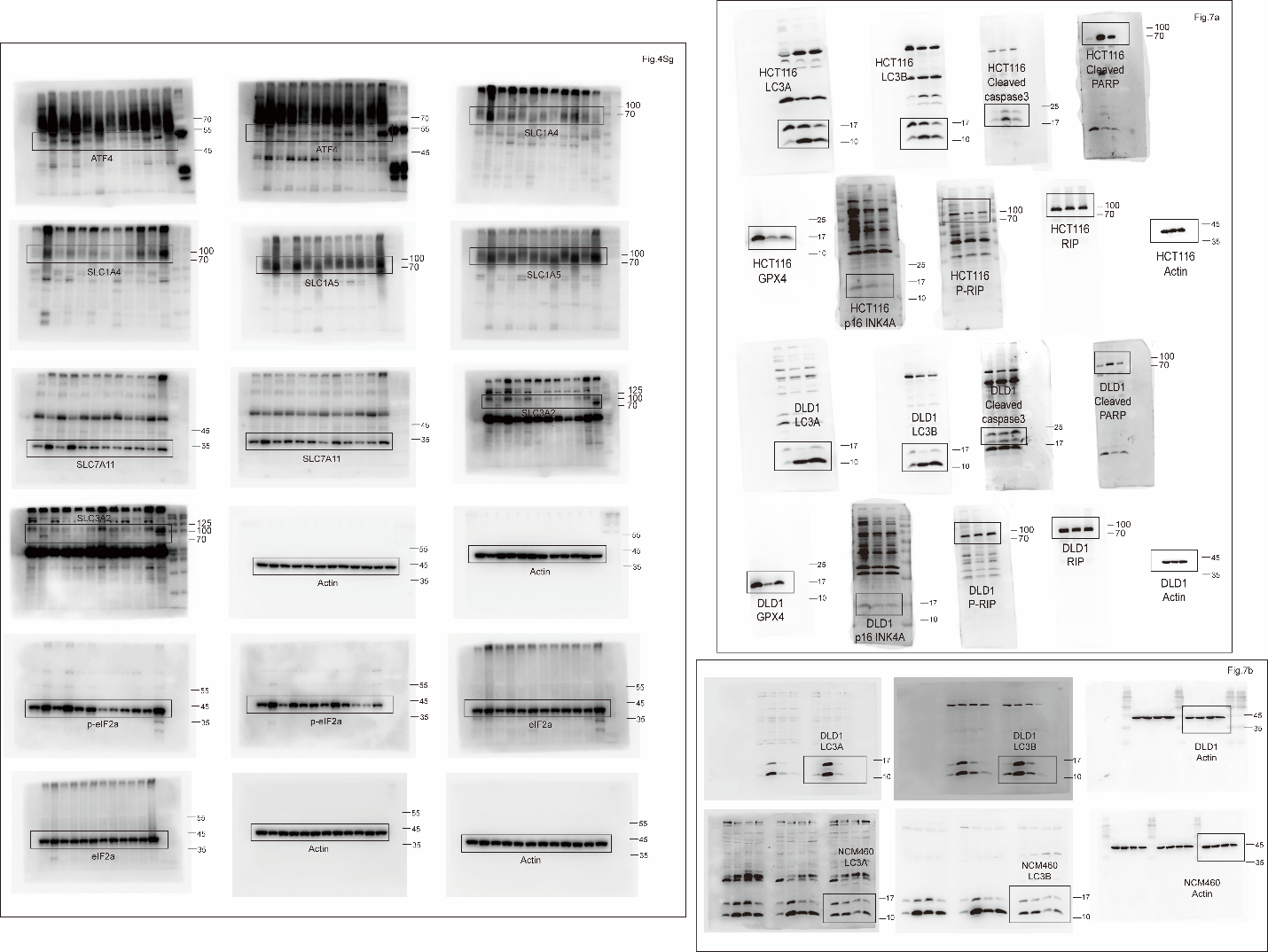


**Fig. S8-2 Original Western blots.**


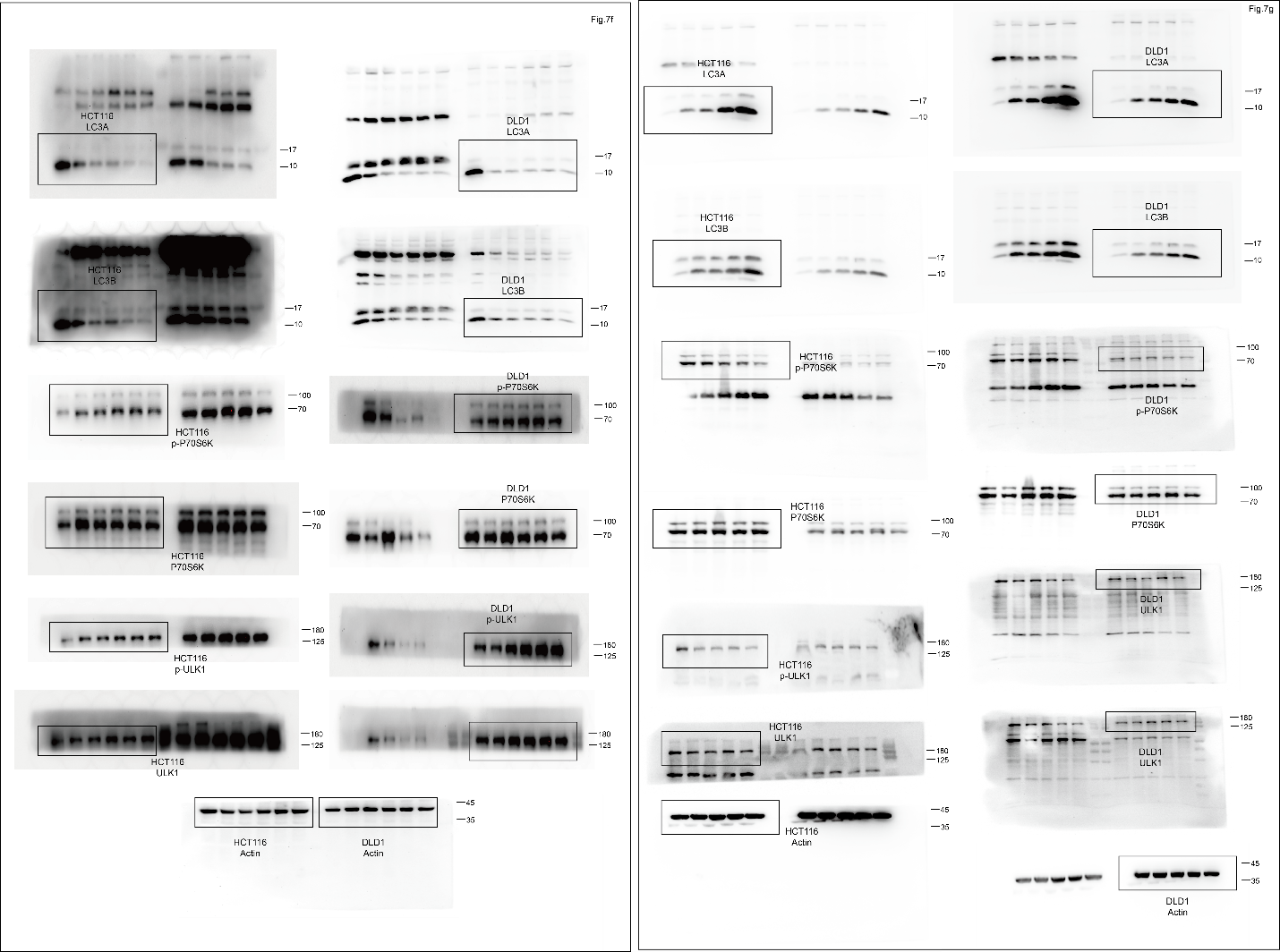


**Fig. S8-3 Original Western blots.**

**Table S1. Identified differential metabolites between colorectal cancer and adjacent normal tissues.**

| Cohort 1 |  | |  | |  | | |
| --- | --- | --- | --- | --- | --- | --- | --- |
| Class | Metabolite | | FC | | OPLSDA-VIP | | |
| Alcohols | Dehydroascorbic acid | | 2.09 | | 2.5 | | |
| Alkylamines | Spermidine | | 1.55 | | 1.9 | | |
| Amino Acids | Taurine | | 1.98 | | 1.8 | | |
| Amino Acids | beta-Alanine | | 3.15 | | 2.2 | | |
| Amino Acids | Arginine | | 0.66 | | 2.5 | | |
| Amino Acids | 3-Sulfinoalanine | | 1.67 | | 2.5 | | |
| Amino Acids | Cysteine | | 1.53 | | 2.8 | | |
| Carbohydrates | Glucose | | 0.20 | | 1.6 | | |
| Carbohydrates | Glycerol | | 0.67 | | 1.7 | | |
| Carbohydrates | Galactose | | 0.39 | | 1.7 | | |
| Carbohydrates | Glyceric acid | | 0.23 | | 1.9 | | |
| Indoles | 5-Hydroxyindoleacetic acid | | 0.22 | | 2.4 | | |
| Inorganic Oxide | Pyrophosphate | | 2.76 | | 2.4 | | |
| Nucleotides | 7-Methylxanthine | | 1.62 | | 1.4 | | |
| Nucleotides | Adenine | | 2.80 | | 1.4 | | |
| Nucleotides | Guanosine | | 3.33 | | 1.5 | | |
| Nucleotides | Guanine | | 1.82 | | 1.6 | | |
| Nucleotides | Thymine | | 2.59 | | 2.0 | | |
| Nucleotides | UMP | | 25.04 | | 2.3 | | |
| Nucleotides | Inosine | | 1.62 | | 2.4 | | |
| Nucleotides | Cytosine | | 1.67 | | 2.6 | | |
| Organic Acids | Phosphoenolpyruvic acid | | 2.34 | | 2.1 | | |
| Vitamins | Ascorbic acid | | 1.90 | | 1.5 | | |
| Vitamins | alpha-Tocopherol | | 0.45 | | 1.7 | | |
| Cohort 2 | |  | |  | |  |  |
| Class | | Metabolite | | FC | | OPLSDA_VIP |  |
| Alcohols | | Lactitol | | 0.64 | | 1.4 |  |
| Alcohols | | Dehydroascorbic acid | | 1.62 | | 1.4 |  |
| Alcohols | | Inositol | | 0.54 | | 1.6 |  |
| Alkylamines | | N-Acetylputrescine | | 9.24 | | 1.7 |  |
| Amino Acids | | Asparagine | | 1.52 | | 1.3 |  |
| Amino Acids | | Aspartic acid | | 1.53 | | 1.5 |  |
| Amino Acids | | Histidine | | 0.54 | | 1.5 |  |
| Amino Acids | | Tyrosine | | 0.48 | | 1.6 |  |
| Amino Acids | | Cystathionine | | 5.64 | | 1.6 |  |
| Amino Acids | | Taurine | | 0.42 | | 1.7 |  |
| Amino Acids | | Pyroglutamic acid | | 1.64 | | 1.8 |  |
| Amino Acids | | beta-Alanine | | 5.83 | | 1.9 |  |
| Amino Acids | | Cysteine | | 1.69 | | 2.0 |  |
| Amino Acids | | Allothreonine | | 2.92 | | 2.2 |  |
| Carbohydrates | | Mannose-6-phosphate | | 0.54 | | 1.4 |  |
| Carbohydrates | | Sorbitol | | 0.49 | | 1.5 |  |
| Carbohydrates | | Coniferin | | 0.41 | | 1.6 |  |
| Carbohydrates | | Melibiose | | 19.21 | | 1.7 |  |
| Carbohydrates | | Galactonic acid | | 0.35 | | 1.7 |  |
| Carbohydrates | | 1,5-anhydroglucitol | | 3.58 | | 1.7 |  |
| Carbohydrates | | Cellobiose | | 2.38 | | 1.8 |  |
| Carbohydrates | | Galactitol | | 0.48 | | 1.9 |  |
| Carbohydrates | | Galacturonic acid | | 0.34 | | 1.9 |  |
| Carbohydrates | | Fructose | | 3.13 | | 2.0 |  |
| Carbohydrates | | Erythrose | | 2.82 | | 2.1 |  |
| Carbohydrates | | Ribose 5-phosphate | | 2.51 | | 2.1 |  |
| Carbohydrates | | Glucose-1-phosphate | | 0.26 | | 2.4 |  |
| Fatty Acids | | Hexacosanoic acid | | 1.61 | | 1.5 |  |
| Fatty Acids | | Pentadecanoic acid | | 0.32 | | 2.0 |  |
| Inorganic Oxide | | Pyrophosphate | | 5.19 | | 1.7 |  |
| Nucleotides | | Ribothymidine | | 1.69 | | 1.3 |  |
| Nucleotides | | Hypoxanthine | | 1.74 | | 1.3 |  |
| Nucleotides | | Adenosine | | 1.84 | | 1.6 |  |
| Nucleotides | | AMP | | 4.34 | | 1.7 |  |
| Nucleotides | | Adenine | | 3.52 | | 1.9 |  |
| Nucleotides | | Dihydrouracil | | 1.73 | | 2.1 |  |
| Nucleotides | | Thymine | | 3.11 | | 2.1 |  |
| Organic Acids | | Hydroxypropionic acid | | 2.84 | | 1.3 |  |
| Organic Acids | | 3,4-Dihydroxybutyric acid | | 0.35 | | 1.3 |  |
| Organic Acids | | Phosphoenolpyruvic acid | | 1.64 | | 1.3 |  |
| Organic Acids | | 3-Hydroxymethylglutaric acid | | 1.94 | | 1.4 |  |
| Organic Acids | | Succinic acid Semialdehyde | | 1.86 | | 1.5 |  |
| Organic Acids | | Ketoleucine | | 0.49 | | 1.8 |  |
| Phenylpropanoic Acids | | 3-Phenyllactic acid | | 1.53 | | 1.4 |  |
| Phenylpropanoic Acids | | Hydroxyphenyllactic acid | | 2.08 | | 1.7 |  |
| Vitamins | | gamma-Tocopherol | | 0.41 | | 1.5 |  |

All the identified differential metabolites satisfy p value<0.05 (T test); |FC|>1.5; VIP>1.2

**Table S2. The MRM parameters for target detection of cysteine related metabolites.**

| Target metabolites | Q1 (Precursor, m/z) | Q3 (Product, m/z) | CE (V) | DP (V) |
| --- | --- | --- | --- | --- |
| L-cysteine | 122 | 59 | 25 | 40 |
| L-cystine | 241.1 | 74.07 | 30 | 60 |
| Taurine | 126 | 108 | 10 | 60 |
| Hypotaurine | 110 | 92 | 10 | 60 |
| Glutathione | 308 | 162 | 21 | 60 |
| GSSG | 613.1 | 355.1 | 31 | 60 |
| Gamma-glutamylcysteine | 251 | 130 | 10 | 60 |
| L-cystathione | 223 | 134 | 13 | 80 |
| L-methionine | 150 | 104 | 13 | 60 |
| S-adenosyl-L-homocysteine | 385.1 | 134.2 | 15 | 40 |
| S-adenosyl-L-methionine | 399.1 | 250 | 15 | 60 |
| L-homocysteine | 136 | 90 | 15 | 60 |
| 2-chloro-L-phenylalanine | 200 | 154 | 30 | 60 |

**Table S3. Primers and oligos used in this study.**

| **Primer name** | **Primer sequences（5’ to 3’）** | | | |
| --- | --- | --- | --- | --- |
| **Primers for** **qRT-PCR** | | | | |
| SLC1A4 | | Forward  Reverse | | TGTTTGCTCTGGTGTTAGGAGT  CGCCTCGTTGAGGGAATTGAA |
| SLC1A5 | | Forward  Reverse | | GAGCTGCTTATCCGCTTCTTC  GGGGCGTACCACATGATCC |
| SLC7A11 | | Forward  Reverse | | TGCTGGGCTGATTTATCTTCG  GAAAGGGCAACCATGAAGAGG |
| SLC3A2 | | Forward  Reverse | | TGAATGAGTTAGAGCCCGAGA  GTCTTCCGCCACCTTGATCTT |
| ATF4 | | Forward  Reverse | | ATGACCGAAATGAGCTTCCTG  GCTGGAGAACCCATGAGGT |
| GSS | | Forward | | GGGAGCCTCTTGCAGGATAAA |
|  |  | Reverse | | GAATGGGGCATAGCTCACCAC |
| HIF1A(HIF-1α) | | Forward | | GAACGTCGAAAAGAAAAGTCTCG |
|  |  | Reverse | | CCTTATCAAGATGCGAACTCACA |
| EPAS1(HIF-2α) | | Forward | | CGGAGGTGTTCTATGAGCTGG |
|  |  | Reverse | | AGCTTGTGTGTTCGCAGGAA |
| β-actin | | Forward | | CATGTACGTTGCTATCCAGGC |
|  |  | Reverse | | CTCCTTAATGTCACGCACGAT |
| **Primers for ChIP-qPCR** | | | | |
| SLC1A4-promoter | | Forward  Reverse | | AAGATCTGAGAGCGTGCTGTTT  ATATCCAAGAAGTGAAATGG |
| SLC1A5-promoter | | Forward  Reverse | | TATTATCTCATTTGAACCCT  TGACAGTGTTGAAATCCTCA |
| SLC7A11-promoter | | Forward  Reverse | | TTGAGCAACAAGCTCCTCCT  CAAACCAGCTCAGCTTCCTC |
| SLC3A2-promoter | | Forward  Reverse | | TTCTCCAGTTTCTCAGGTGT  TTTGATTCAGGAGCAGCTTG |
| **ATF4 binding site for ChIP-qPCR** | | | | |
| SLC1A4 | | TCGTGATGTAACAG (-1462 ~ -1448) | | |
| SLC1A5 | | CCCTTGCAACACTC (-1355 ~ -1341) | | |
| SLC7A11 | | GGCTGATGCAAACC (-378 ~ -364) | | |
| SLC3A2 | | TTGATTCATCATAG (-2318 ~ -2304) | | |
| **Sequence for shRNAs and siRNAs** | | | | |
| SLC1A5  shRNA-1 | | Forward  Reverse | | CCTATGAAGAGAGGAATATCA  TGATATTCCTCTCTTCATAGG |
| SLC1A5  shRNA-2 | | Forward  Reverse | | GCTTATCCGCTTCTTCAACTC  GAGTTGAAGAAGCGGATAAGC |
| SLC7A11 | | Forward | | GCTGAATTGGGAACAACTATA |
| shRNA-1 | | Reverse | | TATAGTTGTTCCCAATTCAGC |
| SLC7A11 | | Forward | | GCTAATTAAAGGTCAAACGCA |
| shRNA-2 | | Reverse | | TGCGTTTGACCTTTAATTAGC |
| ATF4  shRNA-1 | | Forward  Reverse | | GCCTAGGTCTCTTAGATGATT  AATCATCTAAGAGACCTAGGC |
| ATF4  shRNA-2 | | Forward  Reverse | | CCATCTCCCAGAAAGTTTAAC  GTTAAACTTTCTGGGAGATGG |
| shRNA-NC | | Forward  Reverse | | GCTTCGCGCCGTAGTCTTA  TAAGACTACGGCGCGAAGC |
| SLC1A4  siRNA-1 | | Forward  Reverse | | CCUGUUUGCUCUGGUGUUAdTdT  UAACACCAGAGCAAACAGGdTdT |
| SLC1A4  siRNA-2 | | Forward  Reverse | | CGCGGUGUUCAUUGCGCAAdTdT  UUGCGCAAUGAACACCGCGdTdT |
| SLC1A4  siRNA-3 | | Forward  Reverse | | GGUGUGGACAAGAGGAUCAdTdT  UGAUCCUCUUGUCCACACCdTdT |
| SLC1A5  siRNA-1 | | Forward  Reverse | | CUGAGUUGAUACAAGUGAAdTdT  UUCACUUGUAUCAACUCAGdTdT |
| SLC1A5  siRNA-2 | | Forward  Reverse | | GAGGAAUAUCACCGGAACCdTdT  GGUUCCGGUGAUAUUCCUCdTdT |
| SLC1A5  siRNA-3 | | Forward  Reverse | | GUGUUCAUUGCACAGCUCAdTdT  UGAGCUGUGCAAUGAACACdTdT |
| SLC7A11  siRNA-1 | | Forward  Reverse | | GGAAGAGAUUCAAGUAUUAdTdT  UAAUACUUGAAUCUCUUCCdTdT |
| SLC7A11  siRNA-2 | | Forward  Reverse | | GGAAGUCUUUGGUCCAUUAdTdT  UAAUGGACCAAAGACUUCCdTdT |
| SLC7A11 | | Forward | | GCAGCUAAUUAAAGGUCAAdTdT |
| siRNA-3 | | Reverse | | UUGACCUUUAAUUAGCUGCdTdT |
| SLC3A2  siRNA-1 | | Forward  Reverse | | GGAGCUACAGCCUCCUGAAdTdT  UUCAGGAGGCUGUAGCUCCdTdT |
| SLC3A2  siRNA-2 | | Forward  Reverse | | GGUGGAGCUGAAUGAGUUAdTdT  UAACUCAUUCAGCUCCACCdTdT |
| SLC3A2  siRNA-3 | | Forward  Reverse | | GAAUGCUGAGGUUACAGUAdTdT  UACUGUAACCUCAGCAUUCdTdT |
| GSS | | Forward | | CUGUGCAGAUGGACUUCAAdTdT |
| siRNA-1 | | Reverse | | UUGAAGUCCAUCUGCACAGdTdT |
| GSS  siRNA-2 | | Forward  Reverse | | GCAAGAUCCUCUCUAAUAAdTdT  UUAUUAGAGAGGAUCUUGCdTdT |
| EIF2AK3(PERK) | | Forward | | CCGTAGTAAGAAATGGATCAT |
| shRNA-1 | | Reverse | | ATGATCCATTTCTTACTACGG |
| EIF2AK3(PERK)  shRNA-2 | | Forward  Reverse | | GCACACAGATTACAGTCAGAT  ATCTGACTGTAATCTGTGTGC |
| EIF2AK3(PERK)  shRNA-3 | | Forward  Reverse | | CCTCAAGCCATCCAACATATT  AATATGTTGGATGGCTTGAGG |
| HIF1A  siRNA-1 | | Forward  Reverse | | GAAUCAGAAGAUACAAGUAdTdT  UACUUGUAUCUUCUGAUUCdTdT |
| HIF1A  siRNA-2 | | Forward  Reverse | | CCAGCAGACUCAAAUACAAdTdT  UUGUAUUUGAGUCUGCUGGdTdT |
| EPAS1  siRNA-1 | | Forward  Reverse | | AGGUGAAAGUCUACAACAAdTdT  UUGUUGUAGACUUUCACCUdTdT |
| EPAS1  siRNA-2 | | Forward  Reverse | | CAGCUGACAAGGAGAAGAAdTdT  UUCUUCUCCUUGUCAGCUGdTdT |
| **Subcloning primers** | | |  |  |
| pET28a CTH wild-type | | Forward  Reverse | | CCGGAATTCATGCAGGAAAAAGACGCCTC  ACGCGTCGACCTAGCTGTGACTTCCACTTG |
| pET28a CTH E59T | | Forward  Reverse | | GGCCAGCACTCGGGTTTTACATATAGCCGTTCTGGAAATCC GGATTTCCAGAACGGCTATATGTAAAACCCGAGTGCTGGCC |
| pET28a CTH E339V | | Forward  Reverse | | CTAAAGCTATTTACTCTGGCCGTGAGCTTGGGAGGATTCG  CGAATCCTCCCAAGCTCACGGCCAGAGTAAATAGCTTTAG |
| pGL3 SLC1A4  promoter | | Forward  Reverse | | CGGGGTACCGCAGCAAGCAGAGTTGGTGC  CCGCTCGAGGATGCAAACGGGAGCCGCGAAC |
| pGL3 SLC1A5  promoter | | Forward  Reverse | | CGGGGTACCGGCTGGAGTGCAGTGGTGTG  CCGCTCGAGGAGCCCGTTTAGCGGAAGCTG |
| pGL3 SLC7A11  promoter | | Forward  Reverse | | CGGGGTACCCAAGTTCTAGTCATATTTCAAG  CCGCTCGAGAGCTCAGCTTCCTCATGGGC |
| pGL3 SLC3A2  promoter | | Forward  Reverse | | CGGGGTACCTGTAGTCTTGAGAGACTTTC  ACGCGTCGACGGTCGGTGAGCCAGCACGCTC |

| **Table S4 The list of antibodies used in this study** | | |
| --- | --- | --- |
| Rabbit polyclonal antibody anti-ASCT2 | Proteintech | Cat# 20350-1-AP, RRID: AB_2878679 |
| Rabbit monoclonal antibody anti-xCT/SLC7A11 (D2M7A) | Cell Signaling Technology | Cat# 12691, RRID: AB_2687474 |
| Rabbit monoclonal antibody anti-4F2hc/CD98 (D6O3P) | Cell Signaling Technology | Cat# 13180, RRID: AB_2687475 |
| Rabbit polyclonal antibody anti-SLC1A4 | Proteintech | Cat# 13067-2-AP, RRID: AB_2190604 |
| Rabbit polyclonal antibody anti-ATF4 | Proteintech | Cat# 10835-1-AP, RRID: AB_2058600 |
| Rabbit polyclonal antibody anti-GSS | Proteintech | Cat# 15712-1-AP, RRID: AB_2878171 |
| Rabbit monoclonal antibody anti-LC3A (D50G8) XP | Cell Signaling Technology | Cat# 4599, RRID: AB_10548192 |
| Rabbit monoclonal antibody anti-LC3B (D11) XP | Cell Signaling Technology | Cat# 3868, RRID: AB_2137707 |
| Rabbit monoclonal antibody anti-Cleaved Caspase 3 (Asp175) (5A1E) | Cell Signaling Technology | Cat# 9664, RRID: AB_2070042 |
| Rabbit monoclonal antibody anti-Cleaved PARP (Asp214) (D64E10) XP | Cell Signaling Technology | Cat# 5625 RRID: AB_10699459 |
| Rabbit polyclonal antibody anti-GPX4 | Cell Signaling Technology | Cat# 52455, RRID: AB_2924984 |
| Rabbit monoclonal antibody anti-p16 INK4A (D7C1M) | Cell Signaling Technology | Cat# 80772, RRID: AB_2799960 |
| Rabbit monoclonal antibody anti-p-RIP (Ser166) (D1L3S) | Cell Signaling Technology | Cat# 65746, RRID: AB_2799693 |
| Rabbit monoclonal antibody anti RIP (D94C12) XP | Cell Signaling Technology | Cat# 3493, RRID: AB_2305314 |
| Rabbit polyclonal antibody anti-p-p70S6 kinase (Ser371) | Cell Signaling Technology | Cat# 9208, RRID:AB_330990 |
| Rabbit monoclonal antibody anti-p70S6 kinase | Cell Signaling Technology | Cat# 2708, RRID: AB_390722 |
| Rabbit monoclonal antibody anti-pULK1 (Ser757) | Cell Signaling Technology | Cat# 6888, RRID: AB_10829226 |
| Rabbit monoclonal antibody anti-ULK1 (D8H5) | Cell Signaling Technology | Cat# 8054, RRID: AB_11178668 |
| Rabbit polyclonal antibody anti-Actin | Sigma-Aldrich | Cat# A2066, RRID: AB_476693 |
| PERK/EIF2AK3 Polyclonal antibody | Proteintech | Cat#24390-1-AP, RRID:AB_2879521 |
| Phospho-eIF2α (Ser51) (D9G8) XP® Rabbit mAb | Cell Signaling Technology | Cat#3398, RRID:AB_2096481 |
| eIF2α (D7D3) XP® Rabbit mAb | Cell Signaling Technology | Cat#5324, RRID:AB_10692650 |
